# Brain causality alterations in major depressive disorder treatment

**DOI:** 10.3389/fpsyt.2025.1718216

**Published:** 2026-01-27

**Authors:** Madhurima Bhattacharjee, Ioannis Vlachos, Aditi Kathpalia, Jaroslav Hlinka, Martin Brunovský, Martin Bareš, Milan Paluš

**Affiliations:** 1 Department of Complex Systems, Institute of Computer Science of the Czech Academy of Sciences, Prague, Czechia; 2 Department of Electrical and Computer Engineering, Aristotle University of Thessaloniki, Thessaloniki, Greece; 3 Medical School, Aristotle University of Thessaloniki, Thessaloniki, Greece; 4 Clinical Research Program, National Institute of Mental Health, Klecany, Czechia; 5 Third Faculty of Medicine, Charles University, Prague, Czechia

**Keywords:** brain, causality, electroencephalography, major depressive disorder, mutual information

## Abstract

**Introduction:**

Major depressive disorder (MDD) remains a leading cause of disability worldwide, with limited objective biomarkers to guide treatment selection and monitor early therapeutic effects. This study examines whether pharmacological and neurostimulation treatments produce distinct alterations in directional brain connectivity patterns within the first week of treatment and whether baseline connectivity patterns differ between treatment responders and nonresponders assessed at 4–6 weeks.

**Methods:**

We perform an information theory-based causality analysis on instantaneous phase time series derived from electroencephalography recordings of 176 MDD patients. Patients received either pharmacological treatment or neurostimulation and were recorded at baseline (visit 1) and one week post-treatment initiation (visit 2). We quantified directional information flow across 19 EEG channels in five frequency bands ( δ: 1.5-3.5Hz, θ: 4-8Hz, α: 8-12Hz, $\beta_{1}$: 12.5-17.5Hz, $\beta_{2}$: 18-25.5Hz) using global FLOW metrics and local INFLOW/OUTFLOW metrics per channel.

**Results:**

Treatment modalities produced distinct frequency-specific alterations in brain causality. Pharmacological treatment significantly increased global and local information transmission in the *β*_2_ band from visit 1 to visit 2, with widespread effects across brain regions and larger increases in right hemisphere inflow. Neurostimulation treatment increased information flow primarily in the *δ* band, with the strongest effects originating from left hemisphere to the whole brain. At baseline, pharmacological nonresponders exhibited significantly higher *α* band information flow than responders, particularly for right-to-left hemisphere transmission and bilateral inflow. No significant baseline differences emerged between neurostimulation responders and nonresponders, though large effect sizes in *δ* band metrics suggest findings may achieve significance with larger samples.

**Discussion:**

Early-phase directional brain connectivity analysis reveals that pharmacological and neurostimulation treatments engage distinct neural oscillatory mechanisms within the first week. Elevated baseline right-dominant *α*-band causal transmission identifies patients unlikely to respond to pharmacological treatment, enabling alternative interventions before weeks of ineffective therapy. These phase-based causality metrics offer promising biomarkers for early treatment stratification and monitoring. The ability to distinguish treatment responders from nonresponders at baseline and detect treatment-specific changes within one week, before clinical response manifestation, suggests these causality measures capture early mechanistic alterations that could inform timely treatment optimization decisions. (EUDRACT Nos. 2005-000826–22 and 2015-001639-19, registered via www.clinicaltrialsregister.eu).

## 1 Introduction

Major depressive disorder (MDD) is a widespread mental health condition affecting millions of people worldwide (1). It can contribute to various physiological illnesses characterized by enduring unhappiness, loss of interest in activities, and a range of physical and cognitive symptoms. Despite the development of novel treatment strategies for MDD, a critical clinical challenge remains: treatment selection is largely trial-and-error, with patients often enduring weeks of ineffective therapy before alternative approaches are considered. This prolonged period of treatment uncertainty stems from the absence of reliable early biomarkers to predict which patients will respond to specific treatment modalities and to monitor early therapeutic effects before clinical symptom changes manifest (2, 3). The diagnostic criteria for MDD predominantly focus on behavioral aspects and depend on self-reported symptoms, providing limited insight into underlying neurophysiological mechanisms that might guide treatment decisions. There is an urgent necessity for objective biomarkers of MDD to facilitate personalized treatment selection and enable early detection of therapeutic response. Determining efficient, non-invasive diagnostic and therapeutic approaches for MDD poses a significant challenge in clinical neuroscience. Herein two critical clinical questions are addressed: 1) Do pharmacological and neurostimulation treatments produce distinct, detectable alterations in brain connectivity patterns within the first week of treatment before clinical response manifests? 2) Can baseline directional connectivity patterns distinguish patients who will ultimately respond versus not respond to specific treatment modalities assessed after 4–6 weeks through patient self-reporting?

Electroencephalography (EEG) is a widely used, non-invasive neuroimaging technique that measures cerebral electrical activity with a high temporal resolution in the millisecond range, providing benefits over alternative brain imaging modalities. Numerous studies have examined changes in brain functional connectivity, i.e., the pattern of statistical dependencies between remote neurophysiological processes (4), in individuals with MDD, utilizing EEG data (5). However, most EEG analyses typically involves extraction of functional connectivity measures such as correlation, coherence or cordance derived from cerebral signals (6–11). These measures quantify the strength of relationships between brain regions but cannot determine the directionality of information flow. This limitation is significant because understanding which brain regions influence others may be critical for differentiating treatment mechanisms and identifying patients likely to respond to specific interventions. The data obtained from multiple scalp electrodes is analyzed across different frequency bands since these bands are linked to diverse neurophysiological processes and cognitive states. Connectivity patterns within and across these bands provide a more comprehensive and functionally relevant picture of brain dynamics.

Causality analysis addresses this gap by going beyond correlation or coherence to identify cause-and-effect relationships and directional influence patterns in brain networks. In MDD, several studies have already applied directional connectivity methods, including time- and frequency-domain Granger causality, to characterize abnormal information flow in cortico-limbic and fronto-parietal circuits. These approaches have mainly relied on models (typically linear or pre-specified non-linear) and have only rarely been extended to early treatment-related changes in large clinical cohorts. Comparatively little work has used information theory-based causality estimators (like transfer entropy) or estimators operating on phase dynamics to study MDD with EEG, particularly in multivariate set-ups and in the context of treatment prediction and early neurophysiological changes (12–15). The information-theoretic generalization of the Granger causality concept (16) provides a solid mathematical basis for several reliable causality inference methods (17, 18), including the phase dynamics adaptation used in this study (19). Analyzing causality in multi-channel EEG signals might enhance treatment stratification by revealing how different therapies alter the direction and intensity of causal influences among brain regions, thereby illuminating fundamental therapeutic mechanisms.

In this study, we perform a multivariate causality analysis on EEG data recorded from participants diagnosed with MDD and treated with medication or neurostimulation (specifically, low-frequency repetitive transcranial magnetic stimulation [rTMS] or transcranial direct current stimulation [tDCS] targeting the dorsolateral prefrontal cortex). While prior literature has established biomarkers based on non-directional measures, it remains unclear whether directional phase-based causality exhibits similar patterns. To mitigate this gap without bias, we adopt a data-driven approach to address the two aforementioned clinical questions, i.e., of detecting treatment-specific alterations in brain connectivity and of treatment response stratification from baseline connectivity patterns. Early identification of treatment-specific neurophysiological signatures and response-predictive patterns could be particularly advantageous in the initial phase of treatment, as it may facilitate prompt identification of patients unlikely to benefit from their current therapy, enabling timely transition to alternative interventions. Answering these questions could enable earlier, more informed treatment decisions and reduce the trial-and-error period that prolongs patient suffering.

To estimate causality across brain regions, we employ an information-theoretic method called partial mutual information from mixed embedding (PMIME) (19, 20), and examine alterations in brain connectivity and the directional influence among these regions following the initiation of antidepressant treatment. Traditional causality analysis methods such as Granger causality, transfer entropy or partial transfer entropy fail to capture nonlinear relationships in high-dimensional real-world systems. Furthermore, they either assume linear dependencies, are difficult to extrapolate to higher dimensions, are data demanding or are computationally expensive. PMIME addresses these challenges by leveraging mutual information and optimized embedding strategies, and is the only method capable of “general” causality analysis (i.e., no assumption of a model form) in high dimensions. PMIME is a model-free method that is widely applicable for identifying direct causal links between two components in multivariate systems, such as multi-channel EEG signals. This study employs pPMIME, which is a version of PMIME that operates on instantaneous phase time series of EEG data and whose performance has been extensively validated on artificial and real-world data. Phase-based causality analysis effectively reveals genuine causal relationships in weakly linked oscillatory systems, mitigating the effect of noise potentially introduced by amplitude variations. It is a well-known fact that weakly coupled oscillators can alter the phases of each other while their amplitudes remain unchanged (21). We leverage the oscillatory nature of EEG signals and employ phase-based causality analysis to achieve more reliable results. In Section 2 we present the methodology and data used in the analysis. In Section 3 we present the obtained results and then discuss them in Section 4.

## 2 Methods and materials

### 2.1 Phase-based causality analysis (pPMIME)

PMIME/pPMIME (19) is an iterative method that uses forward selection to estimate inherent lagged dependence in coupled multivariate nonlinear dynamical systems. It generates mixed embedding vectors from multiple observed time series of connected subsystems to reveal the causal relationships. The method employs mutual information/conditional mutual information (MI/CMI) I(X;Y)/ I(X;Y| Z) as a selection criterion and uses a statistical test as a stopping criterion of the forward selection procedure. MI measures the average shared information between random variables X and Y, whereas CMI measures the same but conditioned by variable Z. Consider a coupled dynamical system consisting of K subsystems. The time series data for these *K* variables are denoted by $x_{i,\;t}$, where t=1,…,N is the time index and i=1,…,K is the index of the variables. PMIME aims to predict the T-steps ahead future values of a target variable $x_{i}$ by finding a mixed embedding vector ${\text{w}}_{i}=\left[w_{\left(1\right)},w_{\left(2\right)}\ldots \right]$ with an undetermined dimension that maximizes the MI term $I\left(x_{i,\;t+T};w_{i}\right)$. The elements of $w_{i}$ belong to the superset $W=\cup_{i=1}^{K}X_{i}=\{w_{j}\},\;j=1,\ldots ,K\cdot \left(L_{max}+1\right)$, with the sets $X_{i}$ containing the lagged version of each variable, i.e. $X_{i}=\left\{x_{i,\;t},x_{i,\;t-1},\ldots ,x_{i,\;t-L_{\text{max}}}\right\}$, i=1,…,K, where $L_{max}$ is the maximum lag, which can be arbitrarily chosen to be appropriately large. Starting from an empty vector $w_{i}=\;$Ø, the embedding vector is populated sequentially using a forward selection procedure based on the maximization of MI/CMI. The CMI terms for step s of the procedure are of the form $I(x_{i,\;t+T};w^{*}|w_{i}^{s-1})$, where $w_{i}^{s-1}$ is the embedding vector at the previous step ( s−1) and $w^{*}$ is any element of W. The $w^{*}$ that maximizes the CMI term is selected to be added in $w_{i}^{s-1}$ in order to create the new $w_{i}^{s}$. The whole procedure is terminated using a bootstrap resampling test as a stopping criterion, i.e., testing the null hypothesis $H_{0}:I(x_{i,\;t+T};w^{*}|w_{i}^{s-1})=0$. Detailed information regarding the entire procedure can be found in (19, 22). If for a given $I\left(x_{i,\;t+T};w_{i}\right)$, any element of $X_{j}$ is contained in $w_{i}$, then we can interpret that $x_{j}$ causes $x_{i}$. A causality measure can be derived from the following normalized CMI term, bounded between 0 and 1:

(1) $$\text{R}\left(j\to i\right)=\frac{I\left(x_{i,\;t+T};w_{i,\;j}|w_{i,\;rest}\right)}{I\left(x_{i,\;t+T};w_{i}\right)}.$$

In Equation 1, $w_{i}$ is split into elements that belong to $X_{j}$ and all other elements, i.e., $w_{i}=\left[w_{i,\;j},w_{i,\;rest}\right]$ and R(j→i) corresponds to the proportion of information in $x_{i}$ which can be explained by the past values of $x_{j}$ when taking into account the values of the rest of $x_{i}$. PMIME essentially tries to detect which of the present and past values (lags) of $x_{j}$, j=1…K, i.e., $x_{j,\;t},x_{j,\;t-1}\ldots x_{j,\;t-Lmax}$ can be used to optimally predict the future values $x_{i,\;t+T}$, where T can be any value of $T=1,2,\ldots ,T_{max}$.

Similarly, when applied to phase time series ( $\phi_{i,\;t}$), the algorithm (pPMIME in this case) tries to detect which of the present and past lags of $\phi_{j}$, i.e., $\phi_{j,\;t},\phi_{j,\;t-1},\ldots ,\phi_{j,\;t-Lmax}$ for all j, can be used to predict the values of the phase increments ${\text{Δ}}_{T}\phi_{i,\;t+T}=\phi_{i,\;t+T}-\phi_{i,\;t}$. Paluš and Stefanovska demonstrated that, in the bivariate case, ${\text{Δ}}_{T}\phi_{i,\;t+T}$ yields more sensitive causality tests than $\phi_{i,\;t+T}$ itself (23). Equation 1 is modified accordingly using the phase variables.

(2) $${\text{R}}_{\text{P}}\left(j\to i\right)=\frac{I\left(\text{Δ}\phi_{i,\;t+T};w_{\phi_{i},\;\phi_{j}}|w_{\phi_{i},\;\phi_{rest}}\right)}{I\left(\text{Δ}\phi_{i,\;t+T};w_{\phi_{i}}\right)}$$

The phase time series $\phi_{i,\;t}$ can be extracted from the original time series $x_{i,\;t}$ using various methodologies, but herein we employ the commonly used Hilbert transform (HT) method (24, 25). Recently, it was shown that for systems with well-behaving phases or narrowly bandpass filtered data (like the EEG filtered in EEG bands), values of $L_{max}=0$ and T=1 are sufficient to capture causality with reduced computational time (22), so this is adopted herein. With respect to the original pPMIME, we make another simple but intuitive logical change that is critical for mitigating volume conduction effects. Instead of starting from an empty embedding vector, we start with one that includes the $0^{\text{th}}$ lag of the target variable; i.e., if we are creating embedding vectors for $\text{Δ}\phi_{i,\;t+1}=\phi_{i,\;t+1}-\phi_{i,\;t},$ then the embedding vector is initialized as $w_{i}=\left[\phi_{i,\;t}\right]$. This initialization effectively substantially reduces the effect of zero-lag (instantaneous) interactions common in EEG due to volume conduction. Since the CMI criterion only selects source components that provide novel information about the future beyond what is known from the target’s own current state, strong instantaneous correlations (where source and target carry identical information) are recognized as redundant and excluded. This ensures that the derived metrics reflect genuine lagged information flow.

### 2.2 Artificial data and method benchmark

**Figure 1:**
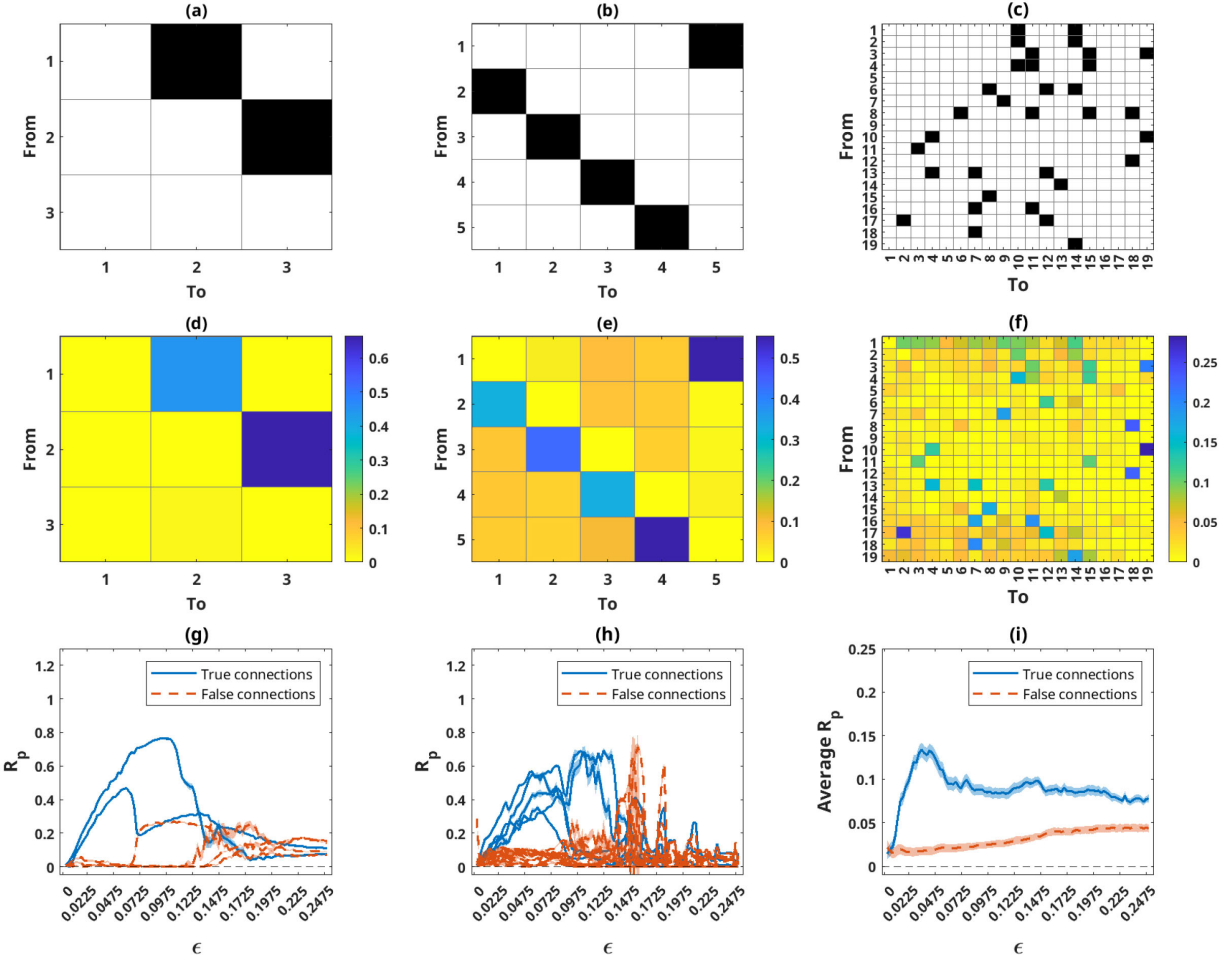
The adjacency matrices for the three cases of coupled Rössler oscillators with *N* = 3, 5, and 19 subsystems in **(a–c)**, respectively, and the corresponding pPMIME causality matrices (R_P_ values) for the coupling strength where the average of all real connections was maximum in **(d–f)**. Average R_P_ values ± standard error (shaded area above and below the average - barely visible in some cases) as a function of coupling strength for the three networks in **(g–i)**. R_P_ values for real connections are plotted in solid blue lines and those for non-connections in orange dotted lines. For clarity, in panel **(i)**, instead of the individual lines, their averages are plotted.

We benchmark the pPMIME method for three cases of basic coupled nonlinear oscillators. We study coupled Rössler systems (26, 27) given by sets of the following equations (one set for each oscillator):

(3) $$\begin{array}{l} {\dot{x}}_{i}=-\omega_{i}\cdot y_{i}-z_{i}+\epsilon \sum_{j=1}^{N}A_{ij}\left(x_{j}-x_{i}\right)\\ {\dot{y}}_{i}=\omega_{i}\cdot x_{i}+a_{i}y_{i}\\ {\dot{z}}_{i}=b_{i}+z_{i}\left(x_{i}-c_{i}\right) \end{array}$$

where i=1,…,N, and N is the total number of subsystems or nodes. The parameters a=0.15, b=0.2, and c=10 were kept constant for all N and were chosen so that all subsystems maintain a stable periodic orbit. The elements of the binary ( 0/1) adjacency matrix A are denoted by $A_{ij}$ and define the connectivity structure of the system, with a value of 1 for $A_{ij}$ indicating a causal connection from subsystem j to i. The parameters $\omega_{i}$ determine the main frequency of oscillation of the $i^{\text{th}}$ subsystem and are set to different values for the subsystems. Finally ϵ is the coupling strength, which we consider to change globally (i.e., it is the same for all subsystems). We studied three cases of 3, 5, and 19 coupled subsystems to validate our method on multi-coupled systems with known directions of influence to imitate real-life scenarios where there are large coupled systems. The values of $\omega_{i}$ were chosen as $\omega_{i}\in \left\{0.95,\;1.00,\;1.05\right\}$ for N=3 (step of $0.05),\;\;\omega_{i}\in \left\{0.90,\;0.95,\ldots ,\;1.10\right\}$ for N=5 (step of 0.05), and $\omega_{i}\in \left\{0.865,\;0.880,\ldots ,\;1.135\right\}$ for N=19 (step of 0.015). For N=3 and 5, we chose the elements of the adjacency matrix such that each node was unidirectionally coupled, forming a serial connection for N=3 and a “ring” of connections for N=5. For N=19, we generated an adjacency matrix randomly such that only 10% of the connections were present. The adjacency matrices for the three cases are displayed in **Figures 1a–c**.

We studied 100 different values of coupling strength ϵ∈{0, 0.0025,…, 0.2475} (step size of 0.0025), and for each one we generated 131072 data points from Equation 3 using the Runge-Kutta (4, 5) method (ode45 in MATLAB ver. R2021a). We chose a sampling time of 0.314 time units, and the first 1000 transient points were discarded. The data were Hilbert transformed, and the first and last 2032 time points were discarded to eliminate any distortion of the data owing to the edge effect of the transform. The remaining data were then divided into 31 segments, with each segment containing 4064 time points. pPMIME and Equation 2 was then applied to each of these segments, which resulted in 31 ( N×N) causality matrices that contained the ${\text{R}}_{\text{P}}$ values for all pairs of i,j. The matrices were then averaged over all 31 segments. Each element in the matrix quantifies the causal influence of a particular node on another node, with the diagonal elements corresponding to the self-influences. For example, the ${\left(i,j\right)}^{\text{th}}$ component of the matrix provides the causal influence of node i on node j. **Figures 1d–f** show causality matrices for the three cases of N, respectively, each for a selected coupling strength for which the average of all real connections was maximum. The specific values of epsilon for the three cases ( ϵ=0.06,0.0625,0.0325 for N=3, 5, and 19, respectively) are small enough to not have widespread synchronization but large enough so that the obtained ${\text{R}}_{\text{P}}$ values are substantial and illustrate the results sufficiently well. As expected, the causality matrices correlate well with the adjacency matrices. **Figures 1g, h**, respectively, show the ${\text{R}}_{\text{P}}$ values for the individual connections for N=3 and N=5, as a function of coupling strength. For the case of N=19, in **Figure 1i**, we show the average of the ${\text{R}}_{\text{P}}$ values corresponding to real connections and of those that correspond to no connections, again as a function of coupling, for reasons of visual clarity. All ${\text{R}}_{\text{P}}$ values plotted are averages across all 31 segments, with error bars showing the standard error of the mean. The real connections are plotted as solid blue lines, and the rest are indicated by orange dotted lines. For all three cases and for low coupling strength values (i.e., prior to the synchronization of the subsystems), the blue lines exhibit monotonically increasing trends and exceed the values of the orange lines. This indicates that the method can accurately capture the direction of causality between the subsystems. Upon synchronization of subsystems, causality cannot be uniquely inferred, and we can see that the orange lines increase and the blue lines decrease. In addition, as the number of subsystems increases, the strength of the detected connections decreases.

### 2.3 EEG data

The study included 176 adult inpatients (age range 18–65 years) with a primary diagnosis of MDD, single or recurrent episode, without psychotic features. The diagnosis of MDD was established according to DSM-IV criteria and confirmed in all participants using the Mini-International Neuropsychiatric Interview (M.I.N.I., Czech version 5.0.0). To ensure at least moderate depressive symptom severity at baseline, participants were required to have a total score on the Montgomery–Åsberg Depression Rating Scale (MADRS) (28, 29) of ≥ 20 and, in the majority of cohorts, a Clinical Global Impression (CGI) score of ≥ 4. The sample predominantly comprised patients with treatment-resistant depression. All participants were hospitalized due to an unsatisfactory response to previous outpatient treatment and fulfilled at least Stage I criteria for treatment-resistant depression according to Thase and Rush (i.e. nonresponse to ≥ 1 adequate antidepressant trial in the current episode). The adequacy of prior antidepressant treatment was verified using the Antidepressant Treatment History Form (ATHF); an ATHF score of ≥ 3 was required to confirm at least 4 weeks of treatment at an adequate dose. A psychotropic medication washout period (median duration 4 days) was implemented for all participants prior to initiation of the new antidepressant treatment. Stringent exclusion criteria were applied to reduce potential confounding, particularly related to concurrent substance use and additional psychopathology. Participants were excluded if they had a current or past history of alcohol or illicit drug abuse or dependence, or any additional Axis I or Axis II psychiatric comorbidity. To minimize pharmacological confounding, individuals with prior exposure to fluoxetine were excluded because of its prolonged biological half-life, and patients who had received electroconvulsive therapy (ECT) within the 3 months preceding study enrollment were not included, given the substantial impact of ECT on EEG measures. More details regarding the sample, recruitment criteria and treatment can be found in previous clinical analysis reports (30–36). Details regarding sex, age, MADRS score, illness duration and response to treatments are shown in **Table 1**. The patients were subjected to either pharmacological or neurostimulation treatments for four weeks. Individuals who showed a ≥ 50% reduction in the MADRS score at the end of the treatment were considered as responders to the treatment. Pharmacological treatment included the following classes of antidepressants: serotonin norepinephrine reuptake inhibitors (SNRI), selective serotonin reuptake inhibitors (SSRI), norepinephrine-dopamine reuptake inhibitors (NDRI), noradrenergic and specific serotonergic antidepressants (NaSSA), and tricyclic antidepressants (TCA). Neurostimulation treatment included low-frequency repetitive transcranial magnetic stimulation (LF-rTMS) at 1 Hz over the right dorsolateral prefrontal cortex (DLPFC) and transcranial direct current stimulation (tDCS) consisted of anodal stimulation over the left DLPFC and cathodal placement over the right supraorbital region, delivered at 2 mA. There were two EEG recording sessions for each patient, one before treatment initiation (visit 1) and a second after one week of treatment (visit 2). Data were recorded with the participants lying in a semirecumbent position with their eyes closed in a maximally alert state. Approximately 10 minutes of data were sampled at 250 Hz or 1000 Hz from 19 electrode channels (Fp1, Fp2, F3, F4, C3, C4, P3, P4, O1, O2, F7, F8, T3, T4, T5, T6, Fz, Cz, and Pz) placed according to the International 10–20 system (37).

**Table 1:** Patient demographics and information. Respondents (R), nonrespondents (NR), number of patients (n).

Characteristics	All Subjects (n=176)	Pharmacological treatment subjects (n=129)		Stimulation treatment subjects (n=47)	
		R (n=68)	NR (n=61)	R (n=16)	NR (n=31)
**AGE**	45.78 ± 11.31	47.58 ± 10.24	43.93 ± 11.83	48.06 ± 11.42	44.29 ± 12.20
**SEX (F:M)**	128:48	54:14	40:21	12:4	22:9
MADRS Score					
Baseline	27.56 ± 3.83	27.23± 3.41	27.90 ± 4.39	25.18 ± 2.04	28.87± 3.68
Final	16.23 ± 8.29	9.04 ± 3.74	22.32 ± 5.33	9.68 ± 2.77	23.41 ± 6.33
% Change	41.52 ± 27.85	66.78 ± 13.00	19.42 ± 17.32	61.50 ± 10.62	19.28 ± 18.12
**Illness Duration** (Weeks)	58.77 ± 73.73	51.72 ± 67.52	66.15 ± 75.94	89.56 ± 107.46	44.10 ± 57.29

Pharmacological subgroups: SSRI (n=46), SNRI (n=63), NDRI (n=14), TCA (n=3), NaSSA (n=3).

Stimulation subgroups: rTMS (n = 26), tDCS (n = 21).

This study was conducted in accordance with the principles outlined in the Declaration of Helsinki and adhered to applicable guidelines for Good Clinical Practice. Ethical approval was obtained from the Ethics Committee of the Prague Psychiatric Centre/National Institute of Mental Health for all study protocols contributing data to this research, with the current retrospective analysis falling under the existing institutional ethics approval for the EEG registry. While a portion of the dataset was derived from clinical trials registered in the EUDRACT database (EUDRACT Nos. 2005-000826–22 and 2015-001639-19, registered via www.clinicaltrialsregister.eu), additional data were obtained from ethically approved studies that were not formally registered in public trial registries. In all cases, adult participants were thoroughly informed about the study procedures and objectives, and they provided written informed consent prior to participation that explicitly authorized the secondary use of their clinical and neurophysiological data for future research purposes.

#### 2.3.1 Preprocessing and causality estimation

The data have been used in previous studies and were preprocessed as described in Ref. (38). In short, the preprocessing included removing extra channels, resampling records to a common 250 Hz sampling frequency, discarding the first and last 30 seconds of each record, re-referencing to the average reference, bandpass filtering the data between 1 and 40 Hz, and removal of data segments with high-power artefacts. The overall aim is to increase the signal-to-noise ratio and make the data more interpretable. More details on the specifics of the preprocessing steps and their individual effects are provided in (38). We further filtered the data into six different frequency bands to focus on causality related to the specific brain activity in each of these bands. The bands were divided as follows: *δ* : (1.5Hz - 3.5Hz), *θ* : (4Hz - 8Hz), *α* : (8Hz - 12Hz), *β*_1_: (12.5Hz - 17.5Hz), *β*_2_: (18Hz -25.5Hz) and *γ* : (26Hz - 40Hz). We implement pPMIME on data segments of 8s duration (2000 time points), as the estimation of CMI has large data requirements. Thus, for each data segment, we estimated six 19 × 19 matrices, one for each band. For each subject, a varying number of segments were available, ranging from 14 to 92. The findings of the *γ* band are not used herein because of R_P_ estimation problems due to improper phase extraction at high frequencies with HT (attributed to the extensive range of the *γ* band).

### 2.4 Flow metrics

**Figure 2:**
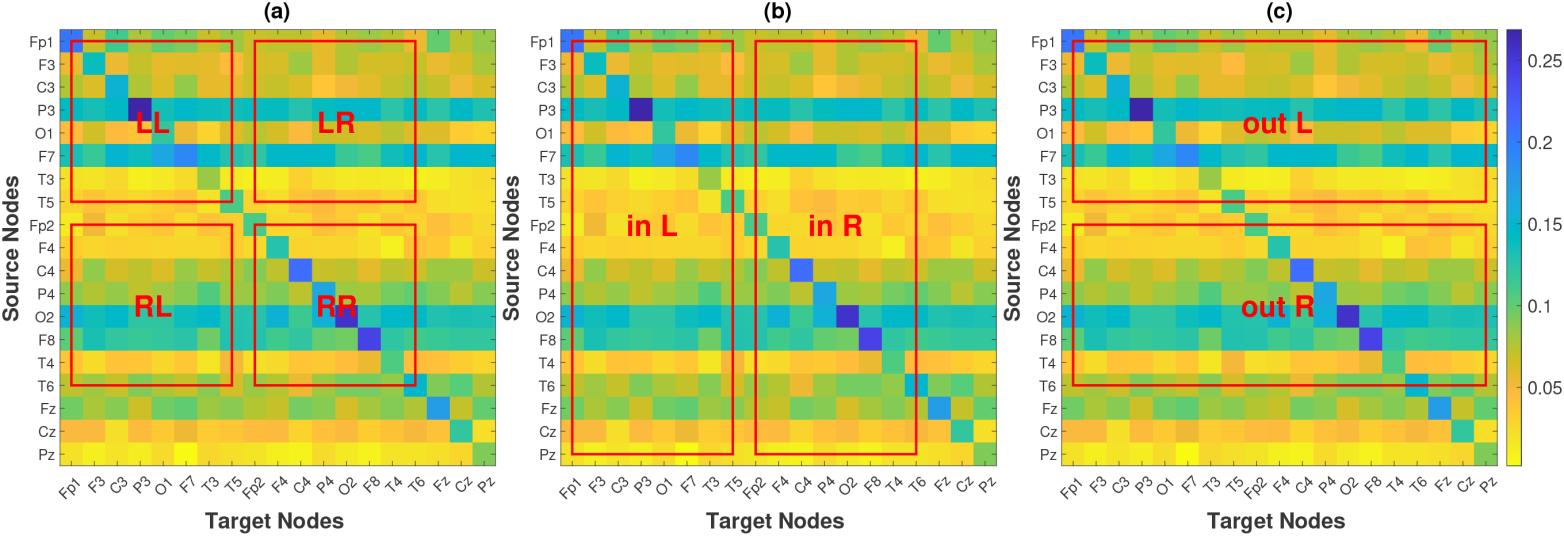
Causality matrix of a subject, along with the regions that are used to define FLOW metrics for hemisphere-to-hemisphere interaction **(a)**, per-hemisphere inflow **(b)** and per-hemisphere outflow **(c)**.

The pPMIME method generates causality matrices of dimensions ( 19×19) for the 19 EEG channels. Each element in the matrix quantifies the causal influence of one channel on another. As detailed in Section 2.1, each entry ${\text{R}}_{\text{P}}\left(i\to j\right)$ is a normalized conditional mutual information term bounded between 0 and 1 that expresses the proportion of phase increment information in channel i that is uniquely explained by the past of channel j, after accounting for the past of i and of all other channels. Because all entries share this common, dimensionless scale, averages of ${\text{R}}_{\text{P}}\left(i\to j\right)$ over anatomically defined sets of connections can be interpreted as expected directional information flow within or between regions. The causality matrices are structured such that the initial eight nodes correspond to channels in the left hemisphere (Fp1, F3, C3, P3, O1, F7, T3, and T5), the subsequent eight nodes correspond to channels in the right hemisphere (Fp2, F4, C4, P4, O2, F8, T4, and T6), and the final three nodes correspond to channels located along the midline of the head (Fz, Cz, and Pz). We define FLOW metrics based on the average of specific parts from a standard causality matrix, as illustrated in **Figure 2**. This allows for the quantification of information flow between brain regions derived from the causal relationships identified in the EEG channel data. We define nine FLOW metrics: The Total Flow ( TF) metric is the aggregate information flow across the 19 channels, calculated by averaging all elements of the matrix. We define 4 metrics for the left hemisphere, LL, LR, out_L, and in_L, which respectively represent the information flow from the left hemisphere to itself, to the right hemisphere, to all the regions of the brain, and the information flow from all regions of the brain to it. Similarly, we define metrics for the right hemisphere, which we denote as RR, RL, out_R, and in_R. The averages are calculated for the regions (excluding the diagonal elements corresponding to the self influences) indicated by the red boxes labeled **LL**, **LR**, **out L**, **in L**, **RR**, **RL**, **out R**, and **in R** in **Figure 2**. Additionally, we study the inflow and outflow for each channel separately, which are defined as ${\text{in}}_{i}=\sum_{j=1}^{19}{\text{R}}_{P}\left(j\to i\right)$, ${\text{out}}_{i}=\sum_{j=1}^{19}{\text{R}}_{P}\left(i\to j\right),\;\forall i\in \left\{1,\;2,\ldots ,\;19\right\}$. So in total, we have 47 metrics. The first 9 are the FLOW metrics, which are global and quantify the information flow of the brain as a whole or per brain hemisphere. The other 38 are the INFLOW and OUTFLOW per channel, which are local metrics and characterize small brain regions that correspond to the positions of the electrodes.

### 2.5 Statistical analysis

After obtaining the causality matrices for each subject over all time segments, visits, and frequency bands, we compute the metrics defined in Section 2.4 for every time segment of each participant. Subsequently, we calculate the mean of the metrics across all the time segments. Thus, for each band and each of the 47 metrics, we have two sets of 176 values (one set for each visit). Since there are multiple factors (response, treatment, and visit) and one of them is a repeated factor (the EEG recording is performed twice on the same patient), we analyzed the data with repeated measures analysis of variance (ANOVA) (39). The repeated measures ANOVA design includes two independent between-subject factors (response and treatment) and one repeated (dependent) within-subject factor (the visit), each with two levels. The response factor has two levels: respondents and nonrespondents, while the levels for the treatment factor are pharmacological and neurostimulation. For the visit, the two levels are visit 1 and visit 2. The setup of the repeated measures ANOVA model includes the individual factors along with all their interactions (3 pairwise and 1 with all three). The analysis for each EEG frequency band is performed separately. Because of the multiple factors and the “rigidity” of the ANOVA design, we do not actually derive statistical conclusions from these p-values; rather, we utilize these results as preliminary information to guide subsequent analysis. Informed by the outcomes derived from the repeated measures ANOVA study, further *post-hoc* analysis for the comparison of individual patient subgroups is performed with Student’s t-tests (independent or paired), and the effect size of any change/difference is estimated by Cohen’s d (40). Non-parametric tests (41) gave similar results to the t-tests, with p-values generally slightly higher with certain differences in statistical significance for some metrics, but no substantial change in the overall conclusions. The ultimate goal of this analysis is to reveal which, if any, of these metrics has the potential to serve as biomarkers.

Our study is exploratory and hypothesis-generating in nature. Given that pPMIME captures non-linear, directional dependencies distinct from the linear correlations found in most prior literature, we opted against restricting our analysis to pre-defined regions of interest. There are no specific hypotheses tested where there is an expected difference based on some prior knowledge. We are conducting a “blind” investigation on which metrics show a difference, and to do this, we employ a comprehensive set of metrics on all possible EEG bands, expecting that most of them will probably not provide statistically significant results. As a matter of fact, we expect our significant results to be constrained to one or, at most, two frequency bands. Furthermore, certain metrics are, by construction, correlated. Because of the high correlation and large number of metrics, global corrections for multiple comparisons are very likely to lead to most p-values being “over-corrected” and rendered insignificant. Since the study is exploratory, over-correcting the p-values is antithetical to the main objective, and in accordance to recent recommendations (42–44), herein we treat our analysis as three separate experiments based on the three metric types (i.e., FLOW, INFLOW, OUTFLOW). Each experiment has its corresponding experiment-wise hypothesis ${\text{H}}_{0}$: *There is no change in the values of any metric of the* sp*ecific type*, along with a “family” of statistical hypothesis tests for the individual metrics of each type. Given that the hypotheses tests inside each family are interchangeable for the corresponding experiment-wise hypothesis (but not interchangeable for the other two experiments) we employ the Benjamini–Yekutieli FDR controlling procedure (45) to obtain adjusted p-values per metric family and EEG band. All p-values in the following section are FDR adjusted and assessed at the *α* = 0.05 significance level. The original, unadjusted p-values from the t-tests and the p-values from the non-parametric permutation tests are provided in the **Supplementary Materials, Tables 4–7**. Controlling for the effects of age and sex using linear models instead of Student’s t-tests did not substantially change our statistical results, neither quantitatively nor qualitatively.

## 3 Results

### 3.1 Results from repeated measures ANOVA

**Figure 3:**
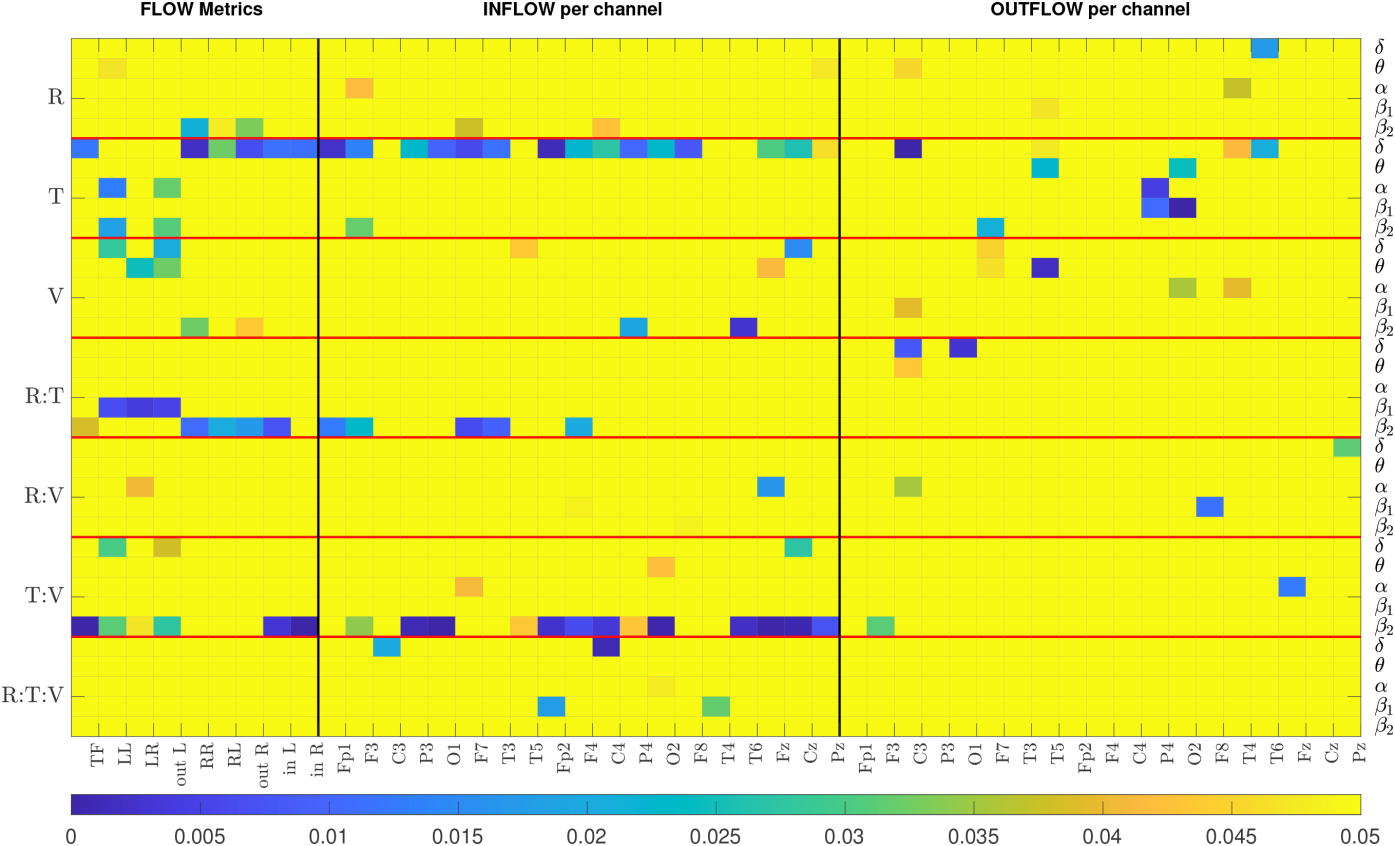
The colormap displays p-values from the repeated measures ANOVA. All p-values above 0.05 are highlighted in yellow. R, Response; T, Treatment; V, Visit. Each row represents the results of a particular band. There are seven blocks corresponding to each factor, separated by a horizontal red line. The metrics corresponding to FLOW, INFLOW and OUTFLOW per channel are separated by a black vertical line.

The results of the repeated measures ANOVA are graphically presented in **Figure 3** and summarized in **Table 2**. The figure displays a colormap highlighting the p-values for the factors and interactions with *p<* 0.05 for each metric. The colormap is organized vertically in seven blocks separated by red horizontal lines. Each block corresponds to a factor or interaction of the factors, and within the block are the five frequency bands. The horizontal axis displays the metrics. The three families of metrics are separated by black vertical lines. The p-values ≥ 0.05 are all set to yellow, while the colder colors correspond to lower p-values. The text around the colormap denotes factors, bands, and metrics. For example, for the TF metric, the treatment factor is significant in the δ band ( p=0.012) and is presented in the first column, the first value of the second block. For the same metric, the only other p-values that are below 0.05 are the response-treatment and treatment-visit interactions in the $\beta_{2}$ ( p=0.038, p=0.0001, respectively). **Table 2** summarizes the repeated measures ANOVA results by presenting the number of metrics/bands that provide a significant p-value for each factor/interaction.

**Table 2:** Total number of metrics/band combinations that provide significant p-values for different factors and their interactions.

Factors	FLOW	INFLOW	OUTFLOW	Total
Response	4	4	4	12
Treatment	10	16	10	36
Visit	6	5	6	17
Response : Treatment	8	5	3	16
Response : Visit	1	3	3	7
Treatment : Visit	8	16	2	26
Response : Treatment : Visit	0	5	0	5

In **Figure 3**, we observe that the majority of p-values that are less than 0.05 are mostly for the δ and the $\beta_{2}$ bands, and that INFLOW metrics show more cases of statistical significance than the OUTFLOW metrics. The summarized repeated measures ANOVA results from **Table 2** show that the three-way interaction factor is not significant for the majority of the metrics and nearly all bands. Only the INFLOW exhibits significant p-values for this interaction for just five metrics spread across all bands. The pairwise interactions between the treatment factor with response and the treatment factor with visit yield the highest number of significant p-values (16 and 26, respectively). On its own, the treatment factor yields 36 statistically significant metric/band combinations. These results indicate that the treatment has the most significant effect on the values of the metrics. Thus, the results suggest analyzing the data from the two treatments independently. The single factors of response and visit give significant values for 12 and 17 cases, but their interaction gives 7, and most of them have relatively high p-values (5 out of the 7 are around 0.04 and the 2 around 0.015). This indicates that even though there is a change in the metric values between visits and there exists a difference between those that responded to treatment and those that did not, these two factors are relatively independent. That is, changes in metric values between visits 1 and 2 are not much related to treatment outcome (response). Combining this with the result for the treatment and visit interaction, we can deduce that treatment may be a strong confounding factor when studying the relationship between response to treatment and metric values. We note that since visit 2 was one week after visit 1, but the respondent/nonrespondent assessment occurred at week 4, it is possible that the week 2 metrics (i.e., brain connectivity) may not have changed enough to “capture” the treatment outcome.

Based on the results of the repeated measures ANOVA, we chose to examine the subjects who received pharmacological treatment and neurostimulation independently. Additionally, we perform two distinct analyses. First, we compare the metrics of the two visits. Here we are not concerned with the treatment outcome but with what effect, if any, the two treatments have on the metrics and consequently on the causal relation in the brain. The second analysis compares the metrics between respondents and nonrespondents to treatment. In this case we compare the metrics of visit 1 for the two response groups. We exclude the visit 2 data for two reasons. First, according to the repeated measures ANOVA results, there is no significant interaction between the response and visit interactions. Second, we cannot be sure about the therapeutic state of the patients at visit 2. It is possible that for some subjects the treatment has produced a change in brain connectivity related to therapy response, while in others it may not have. As a matter of fact, analysis of the visit 2 data alone produced results similar to that of visit 1, but a bit worse, further strengthening the idea that the week 2 brain connectivity changes may not be indicative of the outcome for all subjects.

In the rest of the manuscript we only present results for the FLOW and INFLOW metrics. The ANOVA results for the OUTFLOW metrics show that the statistically significant results for the cases of interest are either much fewer than the INFLOW metrics (2 versus 16 for the treatment/visit interaction) or spread across bands (e.g., compare **Figure 3** results for INFLOW and OUTFLOW for the treatment factor). For OUTFLOW, a minimum of one and a maximum of four channels were sporadically significant in a particular band, whereas all other channels had high p-values. All numerical values of the repeated measures ANOVA p-values are presented in the **Supplementary Materials (Tables 1–3)** for the sake of completeness.

#### 3.1.1 Effect of treatment on brain causality

**Figure 4:**
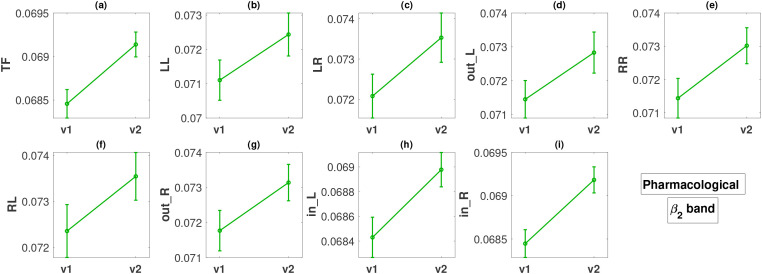
Change in metric values between visit 1 (v1) and visit 2 (v2) in the *β*_2_ band for the pharmacological treatment. The y-axis of all panels **(a–i)** displays the metric values averaged over all subjects along with the standard errors.

**Figure 5:**
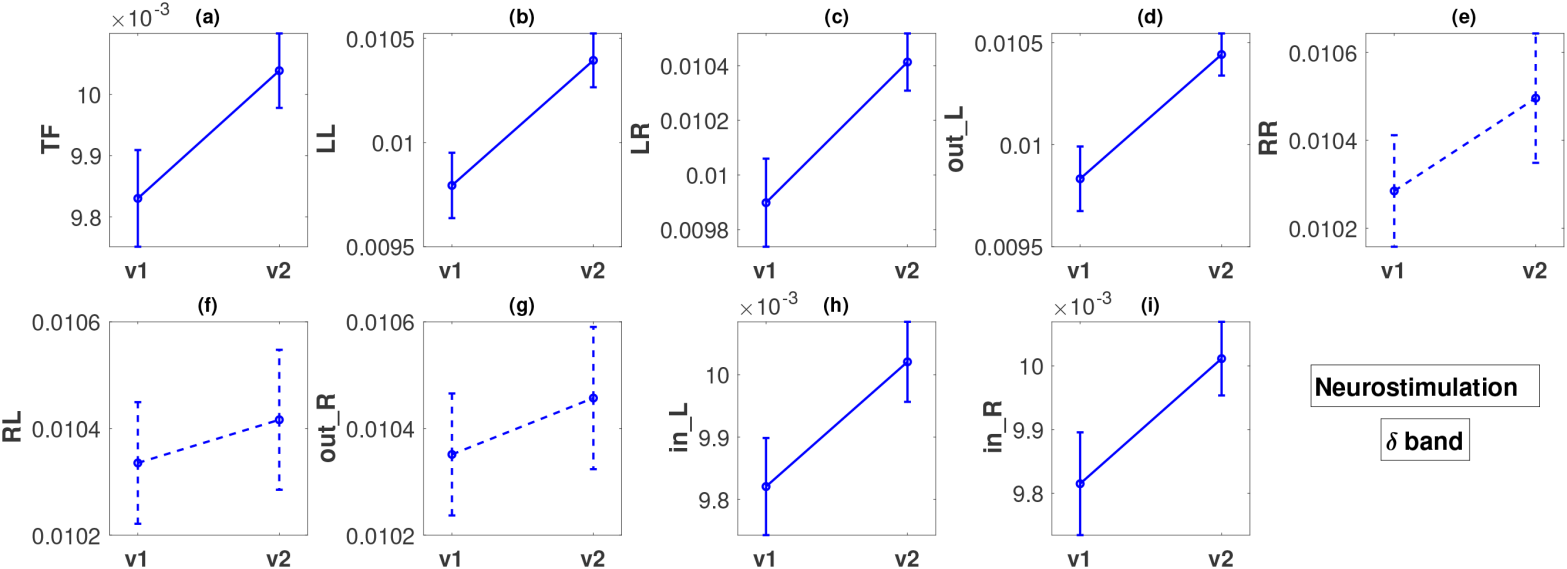
Descriptions of all panels **(a–i)** are similar to **Figure 4**, but for the neurostimulation treatments and the *δ* band. The dashed lines in panels **(e–g)** indicate that there was no statistically significant difference in these cases.

This section presents the results of the change in metrics between visits 1 and 2 for the two therapy groups independently. We perform Student’s t-test (paired) to compare the metric values from two visits. The p-values for all FLOW metrics and all five bands are listed in **Table 3**. The $\beta_{2}$ and the *δ* bands are primarily significant for the FLOW metrics in the pharmacological and neurostimulation groups, respectively. In the first group, the p-values in the $\beta_{2}$ band indicate a substantial difference between visits across all FLOW metrics. In the second treatment group *TF*, *LL*, *LR*, *out_L*, *in_L*, and *in_R* are significantly different in the *δ* band. **Figure 4** illustrates the variation in all the FLOW metrics between the two visits for the pharmacological group in the $\beta_{2}$ band, and **Figure 5** presents the same for the neurostimulation group in the δ. No metric achieved statistical significance after FDR correction in the other three frequency bands; therefore, we omit the presentation of these results graphically (figures corresponding to all metrics and all bands are presented in the **Supplementary Materials, Figures 1–5**). In both figures, the y-axis of the panels displays the metric values averaged over all subjects along with the standard errors of the mean.

**Table 3:** P-values for the comparison of the FLOW metrics between visits 1 and 2 for the two treatment groups. p-values< 0.05 are in bold font.

EEG Band	TF	LL	LR	out_L	RR	RL	out_R	in_L	in_R
Pharmacological: visit 1 vs visit 2									
*δ*	0.931	0.931	0.931	0.931	0.931	0.931	0.931	0.931	0.931
*θ*	0.064	0.312	0.082	0.136	0.500	0.136	0.251	0.064	0.064
*α*	0.718	0.718	0.718	0.718	0.960	0.772	0.895	0.718	0.718
*β*_1_	0.837	0.765	0.765	0.765	0.302	0.302	0.302	0.769	0.967
*β*_2_	*<*0.001	0.049	0.029	0.031	0.023	0.0497	0.031	<0.001	<0.001
Neurostimulation: visit 1 vs visit 2									
*δ*	0.009	0.002	0.006	0.002	0.297	0.551	0.510	0.010	0.010
*θ*	0.916	0.218	0.218	0.218	0.773	0.773	0.773	0.916	0.920
*α*	0.603	0.386	0.386	0.386	0.507	0.507	0.507	0.691	0.507
*β*_1_	0.862	0.761	0.761	0.761	0.761	0.761	0.761	0.862	0.994
*β*_2_	0.229	0.229	0.291	0.229	0.291	0.291	0.291	0.229	0.229

For the pharmacological group, in the $\beta_{2}$ band, all the FLOW metrics (**Figures 4a–i**) show a statistically significant increase from visit 1 to visit 2 with p-values ranging between < 0.001 and 0.049. The smallest p-values are observed for metrics *TF*, *in_L*, and *in_R*, all less than 0.001. The effect size for these three metrics based on Cohen’s *d* is moderate, or close to moderate (*d* = 0.3885, 0.3166, 0.4138, respectively). For the remaining metrics, the effect size is small (*d* in the range of 0.1900 − 0.2429).

In the case of the neurostimulation group and the δ band, most metrics, but not all, show significant changes. The FLOW metrics TF, LL, LR, out_L, in_L, and in_R exhibit a statistically significant increase between the two visits with very low p-values, at a maximum of 0.01 (**Figures 5a–d, h, i**). We note that the smallest p-values ( 0.002) correspond to flows originating from the left hemisphere ( LL and out_L), for which the effect size is again moderate ( d=0.5993,0.6540, respectively) but larger than the effect sizes observed in the pharmacological group. Metrics LR, TF, in_L, and in_R also exhibit a moderate effect size change ( d in the range of 0.4015−0.5434). Contrary to what we observe for the left hemisphere, the metrics corresponding to outflow from the right hemisphere ( RR, RL and out_R) do not exhibit any statistically significant changes (**Figures 5e–g**), with very small effect size ( d in the range of 0.1242−0.2242).

**Figure 6:**
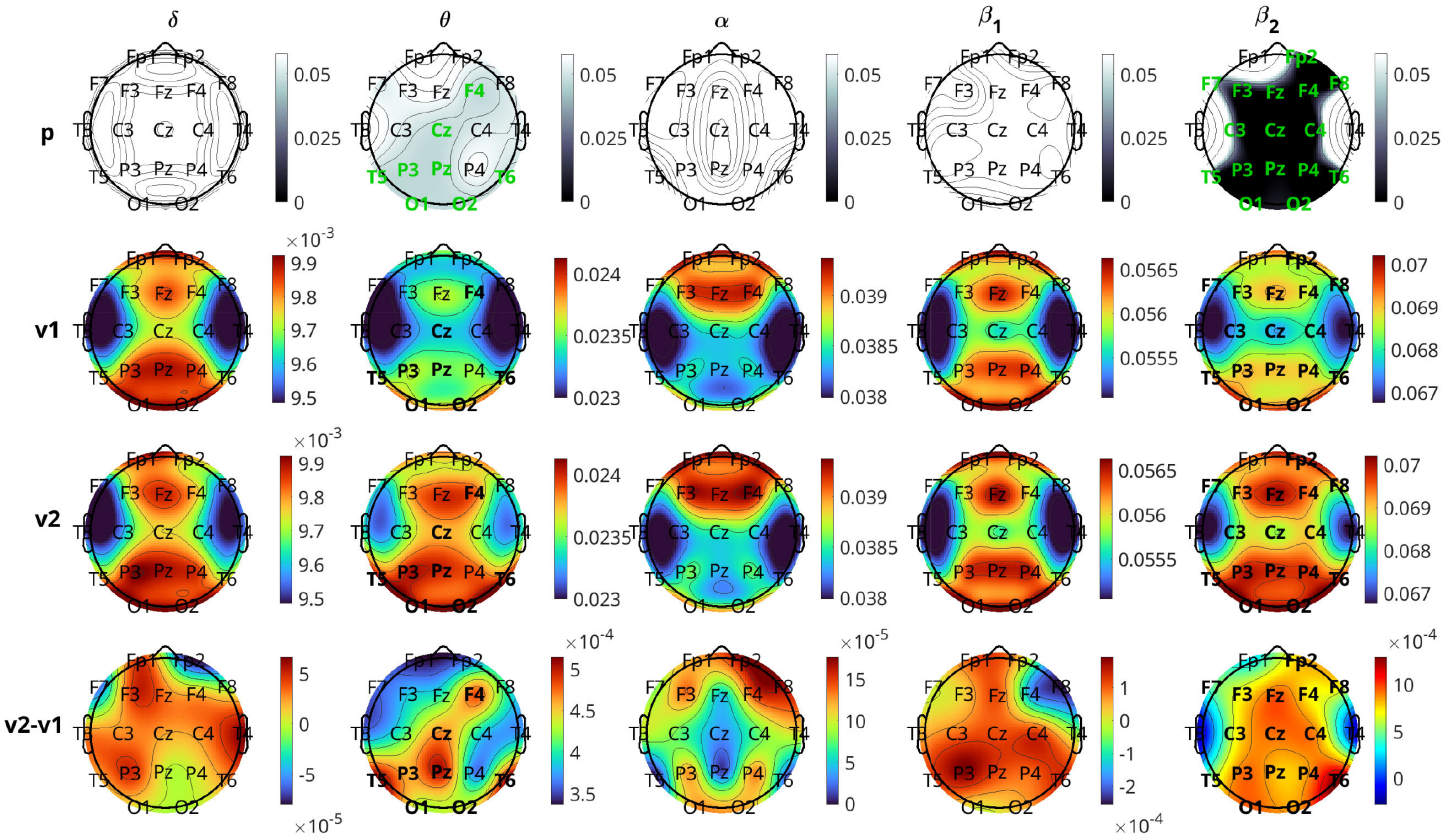
Brain topographical plots for the comparison of inflow per channel between the two visits for the pharmacological group. The first row of panels is the p-values for the comparison. The 2nd and 3rd rows present the average values (across subjects) of the inflow for visit 1 (v1) and visit 2 (v2), while the 4th row presents the difference between the visits. Columns correspond to frequency bands, as indicated by the text above the top panels. Channels with p-values< 0.05 are indicated in green-colored text in the first row.

**Figure 7:**
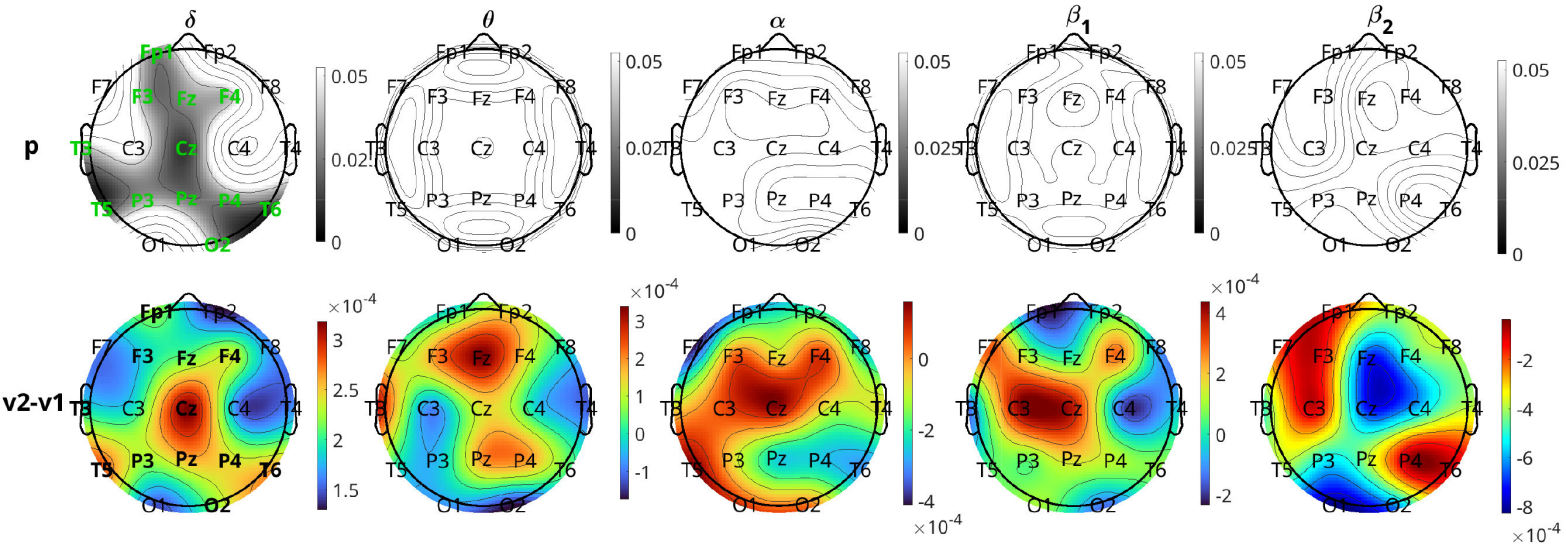
Similar to **Figure 6**, but for the neurostimulation group. Rows of the average inflow per channel for the two visits (2nd and 3rd rows in **Figure 6**) are omitted (given in **Supplementary Materials, Figure 7**).

**Figures 6** and **7** illustrate the results of the change in inflow per channel between the two visits. The results are presented graphically in brain topographical maps and are given numerically in the **Supplementary Materials, Table 5**. In both figures, the first row of panels shows the p-values for the 19 channels in all five bands. The channels that show statistically significant changes in inflow from visit 1 to visit 2 are highlighted in green text. The results for the pharmacological group are presented in **Figure 6**. The second and third rows show topographical maps of the actual values of information flow per channel averaged over all subjects in the five frequency bands for visits 1 and 2, respectively. The fourth row shows the difference in the inflow as visit 2 minus visit 1. The θ and the $\beta_{2}$ bands are the only ones in which the channels had a statistically significant change in inflow. The overall inflow per channel increases from visit 1 to visit 2 in both bands. The channels that are significant for both $\beta_{2}$ and θ bands correspond to the right frontal (F4), central (Cz, Pz), left parietal (P3), posterior temporal (T5, T6), and occipital (O1, O2) regions. Fp2 and P4 are significant only in the $\beta_{2}$ band but still have p-values quite low for the θ band ( p=0.058 both, marginally above 0.05). Comparing the topographic maps of the two visits for both the bands, it can be observed that the $\beta_{2}$ band shows a larger increase in the inflow compared to the θ band. The effect size of the inflow metrics for the significant channels is around moderate ( d in the range of 0.3184−0.5599) for the $\beta_{2}$ band and small for the θ band ( d in the range of 0.1262−0.1872). With respect to localization and the change in inflow, for the $\beta_{2}$ we observe that the statistically significant changes are quite widespread, excluding mainly left prefrontal and mid-temporal regions of the brain. The largest changes (smallest p-values) are observed for the midline frontal (Fz, d=0.4361), parietal (P3, P4, d=0.3871, 0.3824), occipital (O1, O2, d=0.4025, 0.3743), and right posterior temporal (T6, d=0.5599) regions. Comparing the left and right hemisphere p-values for the channels with statistically significant change (F3, C3, P3, O1, F7, T5 *vs* F4, C4, P4, O2, F8, T6), we see that the average p-value in the left is 0.012, while in the right it is 0.002 (almost an order of magnitude difference), indicating that there is more significant change (increase) of inflow in the right hemisphere. Average effect sizes of the significant channels in the left and right hemisphere are 0.3076 and 0.374, respectively.

**Figure 7** presents the results for the neurostimulation group. In this case, the p-values are displayed in the first row of the panels and the difference in the average INLFOW per channel in the second row of panels. The *δ* band is the only band where a significant change in inflow is observed, with p-values of significant channels ranging between 0.013 and 0.040 (Cohen’s *d* in the range 0.2905 − 0.5348). The positive values of the difference in inflow for the *δ* band show that there is a significant increase from visit 1 to visit 2. For the *δ* band, the changes are relatively widespread, including the frontal (Fp1, F3, F4), central (Fz, Cz, Pz), parietal (P3, P4), occipital(O2), and temporal (T3, T5, T6) regions, but the most significant changes, i.e., the lowest p-values, are observed in the central and posterior temporal regions (Cz, T5, and T6 with *p* = 0.013 for all three and *d* = 0.5348, 0.5048, and 0.5007, respectively). Similar to pharmacological treatment, we observed that the average increase in inflow in the significant channels is greater in the right hemisphere (average *d* = 0.4203) than the left (average *d* = 0.3769).

#### 3.1.2 Baseline brain causality difference between response groups

**Figure 8:**
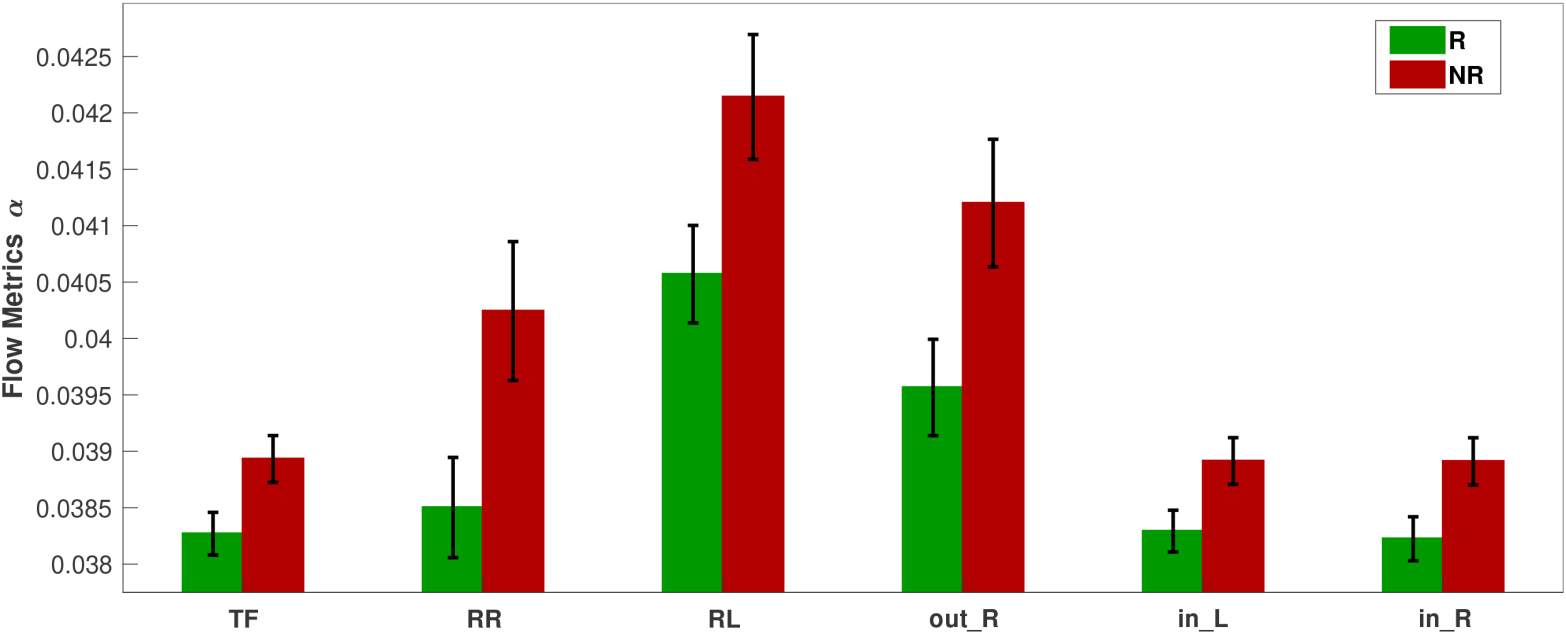
Bar plots of the significantly different FLOW metrics between respondents (R) and nonrespondents (NR) to pharmacological treatment at the time of visit 1 in the *α* band.

**Figure 9:**
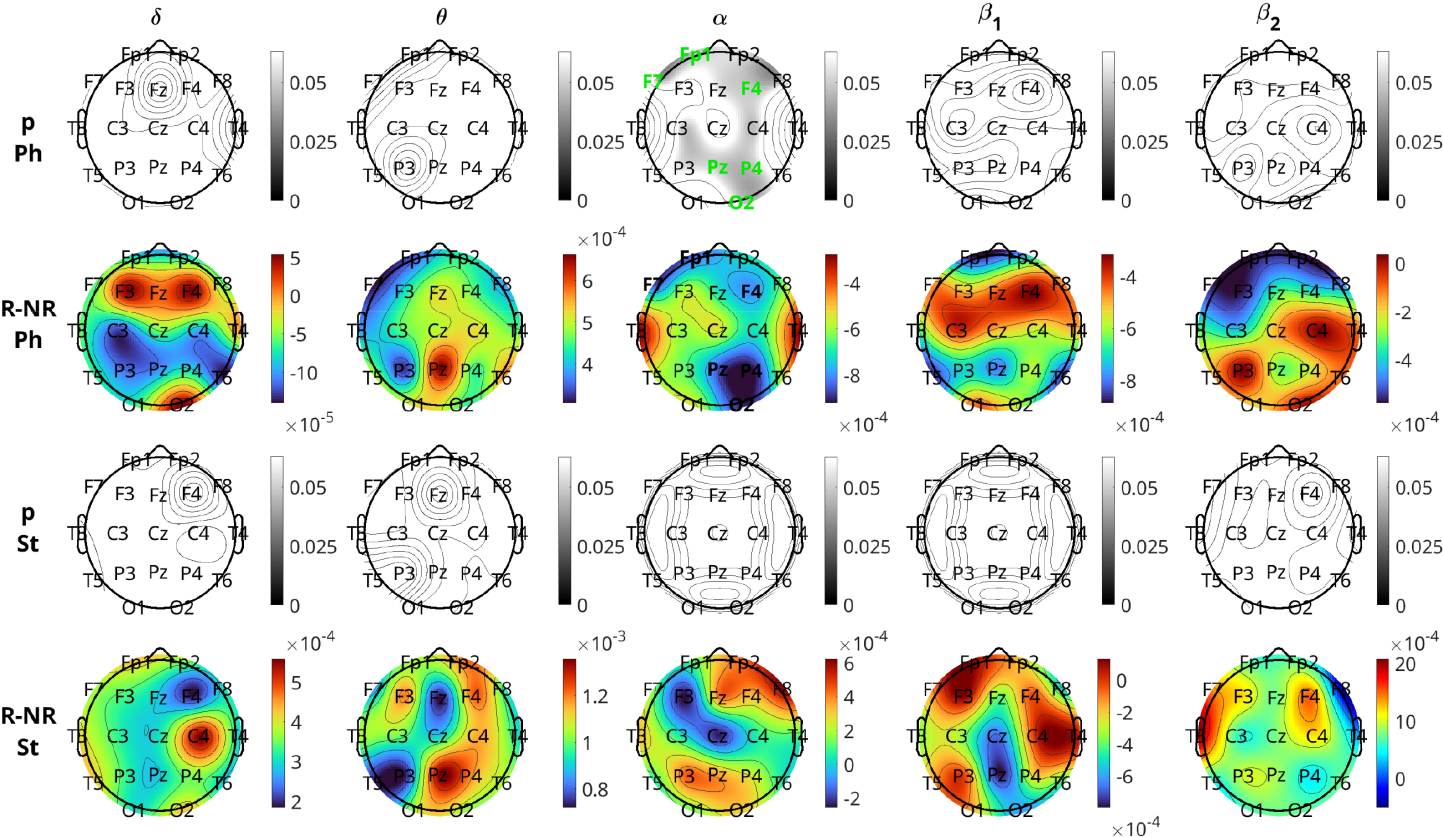
Brain topographical plots for the comparison of inflow per channel between respondents (R) and nonrespondents (NR). The first row of panels is the p-values for the pharmacological (Ph) group, and the second row is the difference in inflow between R and NR averaged over all subjects. The 3rd and 4th rows are the same as the first two but for the neurostimulation (St) group. Columns correspond to frequency bands, as indicated by the text above the top panels. Channels with p-values< 0.05 are indicated in green-colored text in the 1st and 3rd rows.

This section presents the results of the metric analysis with respect to the response to treatment, i.e., the comparison of respondents with nonrespondents. We perform Student’s t-test (independent) to compare the metric values of visit 1 for the two response groups. The p-values for all FLOW metrics and for all five bands are listed in **Table 4**. Any significant difference in the metrics between the response groups will inform us about a difference in brain connectivity patterns of individuals who are going to potentially respond or not to the treatment. From **Table 4**, we observe that only the α band in the pharmacological group shows significant differences for the FLOW metrics TF, RR, RL, out_R, in_L, and in_R with p-values =0.04 for all six metrics (the equality in the p-values is due to the FDR adjustment). For the neurostimulation group, there is no metric that achieves statistical significance. Prior to p-value adjustment there were three metrics in the δ band that showed significant change ( TF, in_L, and in_R with p=0.037, 0.026, and 0.041, respectively), but they did not survive the FDR procedure. The difference of the significant metric values for the pharmacological group in the α band is depicted in **Figure 8**. The y-axis of the figure displays the average over all subjects of the metrics for each response group along with their standard errors. The bar plots indicate that the nonrespondents have higher values for all these metrics than the respondents. The effect size for all these metrics is again at or near the moderate level ( d in the range of 0.3933−0.4215). While statistically significant, these effect sizes indicate a degree of distributional overlap between responders and nonresponders, suggesting that these metrics should be interpreted as group-level mechanistic differences rather than standalone individual diagnostic tools. The bar plots corresponding to all metrics and bands are presented in the **Supplementary Materials (Figures 8–12)**.

**Table 4:** P-values for the comparison of the FLOW metrics at visit 1 between respondents and nonrespondents for the two treatment groups. Significant p-values less than 0.05 are in bold font.

EEG band	TF	LL	LR	out_L	RR	RL	out_R	in_L	in_R
Pharmacological: respondents vs nonrespondents									
*δ*	0.790	0.790	0.790	0.790	0.790	0.790	0.953	0.790	0.790
*θ*	0.516	0.131	0.131	0.131	0.876	0.876	0.876	0.516	0.516
*α*	0.040	0.818	0.688	0.760	0.040	0.040	0.040	0.040	0.040
*β*_1_	0.292	0.292	0.292	0.292	0.292	0.292	0.292	0.292	0.292
*β*_2_	0.914	0.974	0.963	0.968	0.914	0.914	0.914	0.914	0.914
Neurostimulation: respondents vs nonrespondents									
*δ*	0.123	0.531	0.468	0.523	0.468	0.468	0.443	0.123	0.123
*θ*	0.391	0.391	0.391	0.391	0.944	0.944	0.944	0.391	0.391
*α*	0.765	0.765	0.765	0.765	0.765	0.765	0.765	0.777	0.765
*β*_1_	0.851	0.212	0.212	0.212	0.316	0.398	0.367	0.883	0.851
*β*_2_	0.146	0.146	0.119	0.142	0.119	0.119	0.119	0.119	0.244

**Figure 9** depicts the results of the comparison of inflow per channel between respondents and nonrespondents at visit 1 in all five frequency bands. The topographic plots depict the p-values for the comparisons, along with the difference in inflow averaged over all subjects. The first two rows correspond to the pharmacological group, and the last two correspond to the neurostimulation group. The α band is the most significant band for the pharmacological group. The most significant differences between the two response groups can be localized to the frontal (Fp1, F4), left anterior temporal (F7), right parietal (P4), right occipital (O2), and central (Pz) regions (all six having p-values =0.048 and Cohen’s d=0.431 to 0.532, indicating moderate effect size). The channels Fp2, Fz, F8, C3, C4, and P3 had p-values =0.055, which are marginally above the significance threshold. With respect to the nature of the difference, for all the significant channels, the nonrespondents have a higher inflow per channel than the respondents. The $\beta_{1}$ band also had two channels (Fp1 and T5) with p<0.05 before FDR adjustment, but they lost significance after adjustment, and the other three bands had no significant channels.

For the comparison of the response groups in the case of neurostimulation treatment, the results are not as positive. No bands show any statistically significant difference between the response groups for any channel. Prior to the FDR procedure, channels Fp1, T3, T5, C4, and O2 in the δ band had p<0.05, but after adjustment, all of them lost significance with the adjusted p-values of C4 to be 0.082 and the rest higher. The $\beta_{2}$ band also had three channels (F7, T3, and F4), with p-values <0.05 before adjustment, which lost significance afterwards. In both bands, contrary to the pharmacological group, the respondents have a (non-significantly) higher inflow per channel compared to the nonrespondents.

## 4 Discussion

In this study, we conducted an information theory-based causality analysis on EEG data of patients diagnosed with MDD who received pharmacological or neurostimulation treatments. We introduced a slightly adjusted version of a previously developed method (pPMIME) and first tested it on artificial data, where it performed very well in capturing causal relationships for weak coupling. We then used pPMIME to estimate the pairwise strength of the causal relationships between brain locations. To study information flow in the brain, we defined sets of global (FLOW) and local (INFLOW and OUTFLOW per channel) causality metrics. We used repeated measures ANOVA to determine which factors (visit, treatment, response, and all their possible interactions) were the most important. The ANOVA results indicated overall differences between the two treatment groups and that there was no interaction between the response and visit factors. Therefore, we decided to analyze separately the data from the two treatments. We focused on two independent studies: 1) analyzing the impact of each treatment modality on brain causality between visits and 2) comparing baseline/visit 1 metrics for the two response groups.

The FLOW metrics performed well in differentiating the two visits as well as the two response groups, the latter only for the pharmacological treatment. With respect to the local metrics, the OUTFLOW per channel performed much worse than INFLOW. OUTFLOW provided fewer in number and magnitude significant p-values that did not provide any strong localization information. On the other hand, INFLOW provided insight about brain region-specific information flow and indicated brain regions whose “causal behavior” is altered by treatment and may potentially serve as indicators of response to treatment.

A significant consideration in our analysis was the heterogeneity of treatment types within the pharmacological and neurostimulation groups. The pharmacological group comprised five subgroups (SNRI, SSRI, NDRI, TCA, and NaSSA), while neurostimulation included rTMS and tDCS. We performed a sensitivity analysis to test for interaction effects between treatment subgroups and the outcome metrics. Repeated measures ANOVA on the two groups separately followed by FDR adjustment over each family and subsequent *post-hoc* rank based comparison tests revealed that there was no statistically significant difference between the subgroups with respect to causality. While we acknowledge that the absence of statistical significance does not constitute definitive proof of physiological equivalence—given the potential for Type II errors in smaller subgroups—the current analysis did not detect a substantial effect of the heterogeneity. This lack of detectable divergence between the modalities statistically justifies their pooling into a single cohort, consistent with the view that these interventions share convergent downstream effects on neural circuitry. Theoretically, all the different subgroups in pharmacological treatment are known to increase synaptic neurotransmitter availability, such as serotonin, norepinephrine, and dopamine by different mechanisms to prevent the reuptake of the corresponding neurotransmitter into the presynaptic neuron (46). All three neurotransmitters work together to address neurochemical imbalances or neural circuit dysfunction to improve mood, reduce anxiety, and restore emotional equilibrium, thus converging on common downstream circuit-level effects. Similarly, both rTMS and tDCS aim to restore balance in prefrontal–limbic circuitry, albeit through different neurophysiological mechanisms—modulating neurotransmitter release, metabolism, or gene expression but achieving convergent network-level outcomes (47, 48). rTMS (1 Hz right DLPFC): provides inhibitory modulation of overactive right prefrontal regions, indirectly facilitating left prefrontal activity and rebalancing hemispheric asymmetry associated with depression. tDCS (anodal left DLPFC): enhances excitability and connectivity of hypoactive left prefrontal cortex, also aiming to restore network balance. Thus, the first part of this study focused on the changes of information flow metrics due to the overall effect of the pharmacological versus neurostimulation treatment irrespective of subgroups variations.

After one week of pharmacological treatment, we observed a relatively global increase in information flow in $\beta_{2}$ band without strong hemispheric bias. This finding complements prior findings of elevated β power and phase synchronization post-treatment (49, 50), which enhance signal-to-noise ratio and aligns windows of excitability across distant regions. Together, these changes create stronger and more precisely timed carrier signals that could indicate the observed increase in information flow—quantified by causality analyses. One plausible neurobiological mechanism likely involves restoration of neurotransmitter dynamics in circuits affected by depression. Certain brain regions may exhibit insufficient neurotransmitter activation in MDD, leading to impaired synaptic communication. Pharmacological treatments, such as SSRIs, limit the action of corresponding serotonin reuptake transporters, thereby prolonging the presence of neurotransmitters like serotonin at the synapse. This boosts neuronal signaling and facilitates symptom relief. The resulting enhancement in circuit-level communication may manifest as increased information flow, as observed from visit 1 to 2 in the $\beta_{2}$ band for the metrics corresponding to all brain regions. Regionally, the largest inflow increases occurred in the $\beta_{2}$ band, with maximal effects at the midline frontal, parietal, and occipital sites. Since MDD is known to disrupt large-scale cortical and subcortical networks spanning multiple lobes of the brain (51, 52), these results suggest treatment exerts its strongest influence on these interconnected systems. Notably, at visit 2, inflow to local sites in the $\beta_{2}$ band increased more in right hemisphere regions compared to the left. This pattern supports the view that the right hemisphere is affected more in MDD (53) and suggests that treatment may enhance left hemisphere’s capacity to send regulatory signals to the right.

After one week of neurostimulation treatment, the δ band distinguished the two visits by showing a statistically significant increase in global information flow from the left hemisphere to the rest of the brain. This pattern aligns with prior findings that focused on frontal channels and reported stimulation-induced changes in δ band connectivity (54). Regionally, the effects were widespread, with the largest differences observed in the central and posterior temporal areas, alongside significant alterations in the frontal sites—including the left frontopolar, bilateral dorsolateral prefrontal, and midline frontal sites. Physiologically, rTMS and tDCS modulate synaptic plasticity and neural excitability, thereby reshaping functional connectivity by strengthening beneficial neural pathways and weakening maladaptive ones (47, 48). Both approaches excite the hypoactive left prefrontal cortex, which is reflected in the marked increase in left-to-whole-brain information flow in the *δ* band from visit 1 to 2. Notably, FLOW metrics for both hemispheric outflow showed opposite trends from visit 1 to 2 (**Supplementary Materials, Figures 1–5**) across most frequency bands, except for δ, where right-hemisphere increases were present but substantially smaller in effect size compared to the left. This asymmetry aligns with the therapeutic mechanisms of stimulation, which act by selectively enhancing or suppressing neural connections to restore balanced communication patterns, reflected as an increase and decrease in information flow. Unlike the pharmacological group, where nearly all metrics across bands trended upward, neurostimulation produced a more differentiated profile of increases and decreases across regions and frequencies.

The frequency-specific nature of our findings ( $\beta_{2}$ for pharmacological, δ for neurostimulation) suggests that different treatments engage distinct neural oscillatory mechanisms. β oscillations are associated with top-down cortical control and GABAergic inhibition (55, 56), while delta oscillations reflect large-scale cortical-subcortical interactions and homeostatic sleep processes (57, 58). This frequency dissociation may explain why some patients respond better to one treatment modality over another, and supports the development of frequency-targeted therapeutic interventions (59, 60).

The baseline analysis at visit 1 revealed that in the *α* band, patients unlikely to respond to traditional antidepressants exhibited greater total information flow—especially right-to-left outflow and bilateral inflow—than potential responders. At the local level, the significant difference were widespread, the frontal, right parietal, left anterior temporal and right occipital regions showed the largest difference, where the nonresponders showed higher inflow. Our agnostic approach revealed patterns that align with, yet extend, established theories. For example, these findings are consistent with prior reports of right hemisphere hyperactivity (53) and elevated *α* band activity in depression, often spanning large portions of the cortex (2, 61). This confirms that our data-driven causality analysis captures known pathological asymmetries while providing novel detail regarding the directionality of these disparate flows. Physiologically, the elevated information flow corresponding to the right hemisphere before treatment might indicate that the brain is attempting to compensate by increasing overall connectivity and information transfer. However, this hyperconnectivity appears to be maladaptive rather than beneficial which causes the brain to work harder but with reduced efficiency in supporting functions such as processing negative emotions, engaging in repetitive thought patterns, and managing automatic bodily functions that the right hemisphere struggles with.

Across individual subjects, all six baseline significant metrics correlated negatively with proportional symptom changes (i.e. percentage change in depression severity score from baseline : $MADRS_{baseline}-MADRS_{final\;week}),$ with Pearson correlation r∈[−0.242,−0.222] whose 95% percentile bootstrap confidence intervals (CIs) (bootstrap resamples: B=5000) excluded zero, two-sided permutation p-values were small ( pPerm∈[0.0046, 0.0126], 5000 permutations), and FDR adjusted p-values across metrics ranged between 0.013–0.019, while left-to-right flow metrics showed near-zero effects and non-significant p values. The narrow, bootstrap-stable CIs and low permutation p-values (**Supplementary Materials, Table 8**) suggest that these associations are robust to sampling variability and therefore plausibly reproducible in cohorts with similar characteristics, although independent validation is still required. These results are consistent with group-level findings, where nonresponders exhibited higher baseline connectivity than responders. Together these results suggest that elevated α band flow particularly right dominant global measures may indicate a less plastic, maladaptive network configuration that limits pharmacological responsiveness. However, the observed correlations are small-to-moderate in magnitude likely too small to serve as standalone clinical decision tools; therefore as a future work we plan to build a prospective predictive modelling that combines these metrics with other clinical and neurophysiological features, evaluated with proper cross−validation and external validation is required to assess clinical utility and generalizability.

Baseline comparisons between responders (n=16) and nonresponders (n=31) to neurostimulation treatment lacked strong statistical significance due to smaller sample size. However, several consistent patterns emerged: responders exhibited higher δ band total flow and bilateral inflow, higher local inflow across channels, and moderate-to-large effect sizes (Cohen’s d=0.6501, 0.6986, and 0.6364, respectively) for the three global metrics and locally ( d in the range of 0.643 - 0.910 for Fp1, T3, T5, C4, O2). These effect sizes exceed those in the pharmacological group and mirror prior reports of elevated baseline δ band activity in stimulation responders (54), suggesting these differences would likely reach significance in larger samples.

The findings of our study complement the existing results in the literature, which have established that several brain regions are affected in MDD, which is reflected in different frequency bands (2). Our frequency-specific findings align with recent MEG evidence showing that beta-band connectivity within inhibitory control networks is particularly relevant for treatment response prediction in MDD (62). The study reported that non-responders exhibited decreased *β* band connectivity in a left-lateralized frontoparietal network centered on superior parietal gyrus and orbitofrontal cortex—regions we also identified as showing altered causality patterns. This convergence across different methodological approaches (MEG coherence *vs*. EEG phase-based causality) strengthens the evidence that frequency-specific network disruptions are fundamental to treatment resistance, arguing for multiband integration in future analyses.

The larger effect sizes for right hemisphere inflow metrics between visits for both treatments suggest that therapies restore hyperactive right-sided circuits by channeling healthier signals from the rest of the brain. The hemispheric specificity of our findings gains additional support from recent findings (62), that while all MDD patients showed right-lateralized network disruptions (potentially reflecting general disease pathology), only non-responders showed additional left-lateralized frontoparietal hypoconnectivity. This pattern of bilateral dysfunction in non-responders versus primarily right-sided alterations in responders suggests that preserved left hemisphere compensatory mechanisms may be crucial for treatment response. The convergent evidence across studies suggests a hierarchical model of network dysfunction in treatment resistance. Primary right-hemisphere disruptions (observed in all MDD patients) may represent core pathophysiology, while secondary left-hemisphere decompensation (seen specifically in non-responders) indicates a failure of compensatory mechanisms (63, 64). While we did not find statistically significant differences in left hemisphere outflow between responders and non-responders, our finding that non-responders exhibited significantly greater total flow, right-to-left flow, and bilateral inflow is consistent with this pattern of dysregulated interhemispheric communication. This bilateral breakdown of top-down control (65, 66) may explain why standard antidepressants, which primarily target monoaminergic systems, fail to restore network function in these patients (67, 68).

These findings have important clinical implications for personalized medicine in MDD. The distinct *α* band baseline signatures in pharmacological nonresponders suggest that EEG-based causality metrics could be incorporated into clinical decision-making algorithms. While single-metric prediction is limited by moderate effect sizes, patients showing high baseline *α* band information flow, particularly from the right hemisphere, display a physiological profile that suggests they might be better candidates for neurostimulation or combined treatment approaches rather than pharmacotherapy alone. This could reduce the trial-and-error period often associated with antidepressant selection, potentially improving patient outcomes and reducing healthcare costs. An important consideration is the temporal dynamics of treatment response. While clinical response was assessed at 4–6 weeks, our brain connectivity changes were measured at one week. This early timepoint may capture initial neuroplastic changes that precede clinical improvement. The observation that visit 2 metrics did not improve prediction over visit 1 metrics suggests that baseline characteristics may be more informative than early treatment-induced changes for predicting ultimate treatment response. Future studies should include multiple intermediate timepoints to map the trajectory of causality changes throughout treatment.

Study limitations include the absence of explicit statistical tests for hemispheric asymmetry metrics and limited statistical power for baseline responder/nonresponder comparisons in the neurostimulation cohort due to small sample size. Future studies should use larger cohorts, include intermediate timepoints to map causality trajectories, and develop prospective predictive models that combine these metrics with clinical and other neurophysiological features.

This study demonstrates that pharmacological and neurostimulation treatments affect brain information flow differently, with frequency-specific signatures ( $\beta_{2}$ for pharmacological, δ for neurostimulation). We observed significant imbalances in brain connection patterns between responders and nonresponders, with distinct profiles for each treatment modality. The *α* band baseline differences in pharmacological treatment indicate pretreatment EEG profiles carry predictive value for treatment response. These information flow metrics can serve as valuable mechanistic indicators augmenting personalized therapeutic approaches in MDD, though further validation in larger, independent cohorts is essential for clinical translation.

## Data Availability

The raw data supporting the conclusions of this article will be made available by the authors, without undue reservation.
