# Supplementary material for: Brain causality alterations in major depressive disorder treatment
Source: Front Psychiatry. 2026 Jan 27;16:1718216. doi: 10.3389/fpsyt.2025.1718216 (PMC12886382; doi:10.3389/fpsyt.2025.1718216)
Supplement: Supplementary file 1 [file DataSheet1.pdf]

Brain Causality Alterations in Major Depressive Disorder Treatment

(Supplementary materials)

Madhurima Bhattacharjee[1], Ioannis Vlachos[1,2,3], Aditi Kathpalia*[1], Jaroslav Hlinka[1], Martin Brunovský[4,5], Martin Bareš[4,5], and Milan Paluš[1]

[1]Department of Complex Systems, Institute of Computer Science of the Czech Academy of Sciences, Prague,Czech Republic
[2]Department of Electrical and Computer Engineering, Aristotle University of Thessaloniki, 54124 Thessaloniki, Greece
[3]Medical School, Aristotle University of Thessaloniki, Thessaloniki 54124, Greece
[4]Clinical Research Programme, National Institute of Mental Health, Klecany, Czech Republic
[5]Charles University, Third Faculty of Medicine, Prague, Czech Republic

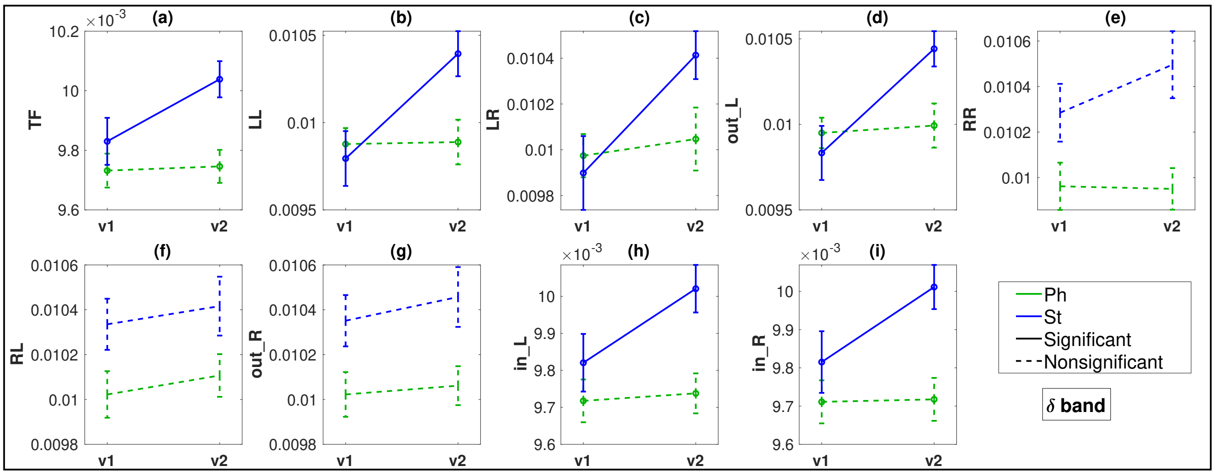

Figure 1: δ band: FLOW Metric comparisons between visits 1 (v1) and 2 (v2) for the pharmacological (Ph) and neurostimulation (St) groups. Metrics with $p < 0.05$ plotted in bold lines and $p \geq 0.05$ are in dotted lines.

*Department of Applied Mechanics and Biomedical Engineering, Indian Institute of Technology, Madras, Chennai, India

Table 1: Repeated measure ANOVA FLOW metric results. Significant p values less than 0.05 are in bold font.

	**TF**	**LL**	**LR**	**out_L**	**RR**	**RL**	**out_R**	**in_L**	**in_R**
					**RESPONSE**				
δ	0.183	0.398	0.656	0.560	0.230	0.667	0.304	0.152	0.212
θ	0.127	**0.047**	0.083	0.062	0.889	0.822	0.805	0.134	0.125
α	0.155	0.796	0.634	0.674	0.127	0.082	0.106	0.143	0.207
β_1	0.113	0.103	0.055	0.072	0.627	0.878	0.771	0.137	0.113
β_2	0.167	0.094	0.114	0.106	**0.021**	**0.048**	**0.033**	0.124	0.182
					**TREATMENT**				
δ	**0.012**	0.186	0.372	0.290	**0.002**	**0.032**	**0.007**	**0.011**	**0.011**
θ	0.123	0.317	0.156	0.198	0.401	0.267	0.319	0.123	0.120
α	0.444	**0.013**	0.064	**0.032**	0.955	0.757	0.812	0.407	0.456
β_1	0.912	0.067	0.116	0.088	0.249	0.173	0.205	0.855	0.855
β_2	0.234	**0.018**	0.055	**0.031**	0.550	0.381	0.468	0.272	0.254
					**VISIT**				
δ	0.099	**0.028**	0.061	**0.020**	0.346	0.510	0.523	0.108	0.155
θ	0.155	0.066	**0.025**	**0.032**	0.829	0.667	0.853	0.160	0.226
α	0.713	0.253	0.269	0.284	0.478	0.595	0.540	0.835	0.649
β_1	0.976	0.402	0.326	0.340	0.750	0.913	0.943	0.929	0.650
β_2	0.191	0.948	0.645	0.924	**0.032**	0.065	**0.044**	0.370	0.082
					**RESPONSE:TREATMENT**				
δ	0.263	0.626	0.972	0.815	0.581	0.424	0.411	0.216	0.278
θ	0.479	0.721	0.720	0.662	0.965	0.929	0.961	0.466	0.479
α	0.288	0.361	0.323	0.362	0.757	0.752	0.713	0.333	0.259
β_1	0.918	**0.007**	**0.004**	**0.005**	0.074	0.101	0.081	0.948	0.889
β_2	**0.038**	0.181	0.070	0.116	**0.011**	**0.020**	**0.017**	**0.007**	0.084
					**RESPONSE:VISIT**				
δ	0.669	0.970	0.973	0.890	0.916	0.722	0.983	0.739	0.512
θ	0.672	0.527	0.219	0.329	0.970	0.948	0.945	0.862	0.536
α	0.196	0.077	**0.040**	0.056	0.508	0.960	0.808	0.196	0.269
β_1	0.267	0.809	0.873	0.837	0.584	0.712	0.710	0.205	0.256
β_2	0.304	0.938	0.521	0.801	0.745	0.613	0.623	0.478	0.137
					**TREATMENT:VISIT**				
δ	0.143	**0.030**	0.153	**0.038**	0.307	0.936	0.781	0.185	0.172
θ	0.260	0.514	0.982	0.754	0.366	0.177	0.254	0.270	0.196
α	0.255	0.794	0.955	0.911	0.554	0.305	0.409	0.322	0.171
β_1	0.734	0.967	0.784	0.800	**0.050**	0.100	0.070	0.668	0.697
β_2	**0.0001**	**0.031**	**0.047**	**0.028**	0.657	0.904	0.773	**0.003**	**0.0001**
					**RESPONSE:TREATMENT:VISIT**				
δ	0.079	0.222	0.050	0.099	0.745	0.333	0.691	0.069	0.085
θ	0.874	0.637	0.994	0.839	0.916	0.821	0.853	0.801	0.983
α	0.283	0.781	0.490	0.649	0.575	0.492	0.501	0.387	0.208
β_1	0.134	0.997	0.846	0.880	0.368	0.545	0.461	0.103	0.124
β_2	0.716	0.488	0.405	0.512	0.793	0.912	0.890	0.586	0.563

Table 2: Repeated measure ANOVA INFLOW per channel metric results. Significant p values less than 0.05 are in bold font.

	Fp1	F3	C3	P3	O1	F7	T3	T5	Fp2	F4	C4	P4	O2	F8	T4	T6	Fz	Cz	Pz
**RESPONSE**																			
δ	0.153	0.208	0.595	0.164	0.320	0.126	0.093	0.105	0.474	0.371	0.158	0.136	0.067	0.198	0.244	0.730	0.408	0.104	0.321
θ	0.133	0.105	0.125	0.218	0.155	0.156	0.089	0.156	0.137	0.103	0.101	0.086	0.259	0.126	0.139	0.129	0.196	0.174	**0.048**
α	0.126	**0.042**	0.146	0.239	0.225	0.062	0.664	0.256	0.398	0.191	0.221	0.161	0.129	0.375	0.734	0.081	0.105	0.072	0.205
β_1	0.216	0.221	0.173	0.129	0.122	0.237	0.077	0.120	0.116	0.165	0.207	0.087	0.116	0.128	0.203	0.075	0.168	0.056	0.054
β_2	0.376	0.473	0.316	0.091	0.228	**0.038**	0.392	0.281	0.530	0.169	**0.042**	0.334	0.130	0.936	0.377	0.214	0.192	0.868	0.228
**TREATMENT**																			
δ	**0.002**	**0.014**	0.140	**0.023**	**0.010**	**0.006**	**0.011**	0.201	**0.001**	**0.023**	**0.027**	**0.010**	**0.023**	**0.008**	0.064	0.186	**0.030**	**0.026**	**0.046**
θ	0.096	0.064	0.132	0.222	0.189	0.101	0.073	0.222	0.140	0.116	0.091	0.106	0.221	0.099	0.091	0.165	0.151	0.131	0.134
α	0.452	0.392	0.646	0.438	0.339	0.571	0.414	0.338	0.993	0.551	0.375	0.435	0.328	0.776	0.254	0.335	0.877	0.553	0.330
β_1	0.823	0.727	0.915	0.995	0.857	0.854	0.754	0.340	0.837	0.660	0.581	0.715	0.928	0.807	0.532	0.746	0.987	0.896	0.398
β_2	0.302	**0.032**	0.171	0.158	0.197	0.158	0.493	0.142	0.450	0.119	0.138	0.495	0.087	0.112	0.130	0.236	0.082	0.393	0.299
**VISIT**																			
δ	0.135	0.125	0.247	0.093	0.371	0.589	0.173	**0.044**	0.951	0.100	0.541	0.069	0.165	0.346	0.141	0.055	0.127	**0.015**	0.107
θ	0.313	0.112	0.421	0.185	0.348	0.169	0.065	0.102	0.205	0.133	0.378	0.093	0.619	0.229	0.446	0.176	**0.041**	0.086	0.081
α	0.457	0.674	0.756	0.778	0.747	0.131	0.872	0.777	0.748	0.740	0.875	0.504	0.940	0.491	0.966	0.134	0.523	0.634	0.301
β_1	0.478	0.887	0.193	0.808	0.792	0.515	0.567	0.918	0.615	0.830	0.499	0.836	0.779	0.241	0.385	0.705	0.881	0.255	0.568
β_2	0.427	0.217	0.181	0.277	0.581	0.401	0.125	0.382	0.228	0.684	0.526	**0.020**	0.226	0.293	0.447	**0.003**	0.290	0.946	0.269
**RESPONSE:TREATMENT**																			
δ	0.161	0.411	0.463	0.319	0.272	0.232	0.201	0.132	0.328	0.984	0.213	0.110	0.273	0.209	0.573	0.247	0.439	0.487	0.364
θ	0.347	0.409	0.625	0.490	0.582	0.384	0.406	0.567	0.414	0.468	0.549	0.300	0.648	0.401	0.523	0.615	0.633	0.534	0.413
α	0.303	0.507	0.284	0.217	0.404	0.294	0.428	0.584	0.163	0.284	0.232	0.176	0.335	0.099	0.558	0.911	0.255	0.656	0.161
β_1	0.536	0.908	0.861	0.897	0.696	0.913	0.974	0.873	0.743	0.793	0.934	0.997	0.973	0.690	0.865	0.996	0.775	0.633	0.614
β_2	**0.013**	**0.023**	0.099	0.136	0.151	**0.006**	**0.009**	0.065	0.088	**0.020**	0.135	0.295	0.122	0.731	0.331	0.105	0.075	0.879	0.131
**RESPONSE:VISIT**																			
δ	0.708	0.387	0.479	0.580	0.802	0.935	0.665	0.892	0.397	0.721	0.120	0.721	0.420	1.000	0.552	0.527	0.358	0.592	0.755
θ	0.933	0.633	0.692	0.944	0.394	0.909	0.938	0.812	0.381	0.585	0.447	0.761	0.215	0.895	0.723	0.631	0.813	0.592	0.462
α	0.476	0.196	0.370	0.095	0.228	0.247	0.303	0.525	0.461	0.271	0.652	0.517	0.285	0.111	0.988	0.071	**0.017**	0.418	0.340
β_1	0.595	0.151	0.266	0.223	0.059	0.329	0.503	0.562	0.372	**0.049**	0.221	0.153	0.790	0.881	0.180	0.728	0.479	0.432	0.976
β_2	0.199	0.767	0.321	0.560	0.282	0.177	0.088	0.301	0.365	0.800	0.634	0.204	0.266	**0.049**	0.313	0.598	0.133	0.319	0.351
**TREATMENT:VISIT**																			
δ	0.351	0.416	0.403	0.271	0.392	0.107	0.315	0.075	0.287	0.160	0.827	0.075	0.146	0.233	0.634	0.123	0.288	**0.027**	0.089
θ	0.352	0.578	0.121	0.179	0.101	0.386	0.888	0.179	0.400	0.271	0.161	0.631	**0.042**	0.254	0.138	0.189	0.890	0.317	0.364
α	0.141	0.606	0.828	0.251	0.599	**0.041**	0.384	0.748	0.149	0.505	0.454	0.138	0.422	0.078	0.543	0.102	0.311	0.833	0.274
β_1	0.187	0.756	0.468	0.393	0.754	0.514	0.398	0.558	0.607	0.394	0.172	0.742	0.554	0.738	0.606	0.701	0.613	0.601	0.974
β_2	0.600	**0.034**	0.206	**0.001**	**2.27e-05**	0.239	0.474	**0.044**	**0.003**	**0.006**	**0.004**	**0.043**	**3.24e-04**	0.062	0.997	**0.002**	**8.13E-06**	**0.001**	**0.007**
**RESPONSE:TREATMENT:VISIT**																			
δ	0.127	0.435	**0.020**	0.106	0.272	0.098	0.060	0.182	0.126	0.351	**0.001**	0.444	0.258	0.338	0.142	0.115	0.403	0.079	0.198
θ	0.659	0.918	0.695	0.563	0.906	0.568	0.908	0.411	0.918	0.751	0.701	0.599	0.628	0.679	0.812	0.990	0.547	0.668	0.957
α	0.310	0.795	0.869	0.237	0.528	0.180	0.536	0.246	0.371	0.106	0.874	0.340	**0.048**	0.387	0.797	0.085	0.162	0.854	0.372
β_1	0.129	0.110	0.565	0.091	0.094	0.309	0.311	0.067	**0.018**	0.161	0.094	0.347	0.902	0.537	**0.032**	0.247	0.488	0.575	0.294
β_2	0.131	0.700	0.749	0.693	0.610	0.818	0.532	0.116	0.947	0.627	0.867	0.770	0.244	0.563	0.460	0.501	0.546	0.122	0.967

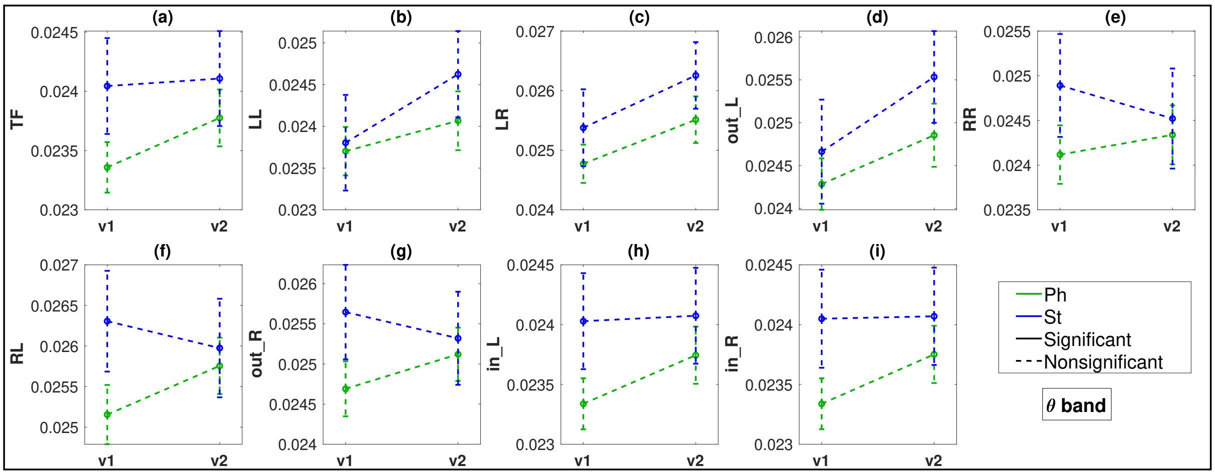

Figure 2: θ band: FLOW Metric comparisons between visits 1 (v1) and 2 (v2) for the pharmacological (Ph) and neurostimulation (St) groups. Metrics with $p < 0.05$ plotted in bold lines and $p \geq 0.05$ are in dotted lines.

Table 3: Repeated measure ANOVA OUTFLOW per channel metric results. Significant p values less than 0.05 are in bold font.

	Fp1	F3	C3	P3	O1	F7	T3	T5	Fp2	F4	C4	P4	O2	F8	T4	T6	Fz	Cz	Pz
**RESPONSE**																			
δ	0.943	0.386	0.152	0.615	0.560	0.752	0.419	0.600	0.634	0.745	0.598	0.982	0.905	0.974	0.864	**0.017**	0.766	0.417	0.547
θ	0.775	0.861	**0.045**	0.242	0.598	0.451	0.066	0.427	0.846	0.869	0.651	0.576	0.781	0.712	0.977	0.402	0.085	0.529	0.942
α	0.748	0.668	0.970	0.176	0.192	0.547	0.597	0.520	0.898	0.691	0.103	0.718	0.328	0.478	**0.037**	0.055	0.166	0.251	0.515
β_1	0.700	0.977	0.975	0.664	0.614	0.950	0.562	**0.047**	0.342	0.894	0.264	0.331	0.086	0.717	0.320	0.459	0.929	0.787	0.967
β_2	0.213	0.696	0.938	0.661	0.556	0.829	0.736	0.106	0.188	0.397	0.914	0.779	0.311	0.360	0.951	0.890	0.854	0.518	0.974
**TREATMENT**																			
δ	0.293	0.587	**1.48E-04**	0.936	0.188	0.389	0.336	**0.048**	0.142	0.418	0.205	0.972	0.576	0.590	**0.042**	**0.021**	0.641	0.689	0.136
θ	0.436	0.598	0.067	0.681	0.648	0.540	0.952	**0.023**	0.578	0.745	0.949	0.561	**0.025**	0.336	0.776	0.052	0.079	0.337	0.404
α	0.219	0.485	0.472	0.922	0.560	0.617	0.502	0.336	0.131	0.628	0.449	**0.004**	0.678	0.251	0.437	0.981	0.861	0.953	0.094
β_1	0.558	0.701	0.979	0.912	0.152	0.130	0.074	0.632	0.681	0.418	0.451	**0.011**	**1.55E-04**	0.097	0.258	0.563	0.123	0.874	0.897
β_2	0.836	0.832	0.319	0.830	0.874	**0.021**	0.258	0.119	0.330	0.332	0.628	0.064	0.100	0.070	0.191	0.901	0.786	0.379	0.638
**VISIT**																			
δ	0.307	0.581	0.899	0.202	0.498	**0.045**	0.854	0.075	0.390	0.356	0.776	0.987	0.278	0.243	0.256	0.771	0.382	0.073	0.479
θ	0.127	0.599	0.577	0.051	0.060	**0.047**	0.577	**0.002**	0.479	0.383	0.803	0.900	0.430	0.635	0.739	0.442	0.356	0.240	0.889
α	0.343	0.606	0.885	0.905	0.060	0.543	0.646	0.511	0.555	0.574	0.679	0.225	**0.036**	0.968	**0.040**	0.318	0.485	0.693	0.217
β_1	0.524	0.325	**0.039**	0.640	0.760	0.087	0.149	0.223	0.642	0.849	0.523	0.930	0.631	0.763	0.868	0.468	0.575	0.932	0.116
β_2	0.635	0.708	0.245	0.810	0.877	0.243	0.344	0.405	0.595	0.917	0.205	0.577	0.514	0.397	0.075	0.590	0.163	0.368	0.121
**RESPONSE:TREATMENT**																			
δ	0.341	0.654	**0.008**	0.249	**0.003**	0.982	0.438	0.246	0.322	0.109	0.690	0.194	0.546	0.386	0.296	0.282	0.225	0.265	0.785
θ	0.200	0.345	**0.043**	0.052	0.999	0.254	0.184	0.878	0.342	0.298	0.925	0.318	0.902	0.653	0.415	0.693	0.065	0.182	0.800
α	0.526	0.806	0.388	0.208	0.496	0.820	0.244	0.951	0.633	0.585	0.947	0.414	0.630	0.804	0.749	0.707	0.291	0.663	0.290
β_1	0.126	0.351	0.988	0.365	0.187	0.663	0.617	0.197	0.817	0.651	0.871	0.737	0.718	0.068	0.442	0.799	0.856	0.182	0.950
β_2	0.055	0.186	0.674	0.725	0.811	0.996	0.540	0.788	0.719	0.306	0.375	0.124	0.912	0.068	0.063	0.316	0.534	0.182	0.827
**RESPONSE:VISIT**																			
δ	0.925	0.209	0.748	0.221	0.630	0.850	0.539	0.977	0.782	0.630	0.234	0.697	0.883	0.655	0.420	0.565	0.095	0.374	**0.031**
θ	0.479	0.409	0.977	0.433	0.443	0.378	0.444	0.871	0.681	0.227	0.493	0.739	0.797	0.063	0.464	0.467	0.243	0.341	0.813
α	0.704	0.389	**0.035**	0.905	0.426	0.774	0.072	0.695	0.280	0.438	0.951	0.591	0.245	0.894	0.201	0.750	0.141	0.733	0.551
β_1	0.656	0.326	0.202	0.569	0.756	0.669	0.802	0.764	0.710	0.440	0.851	0.875	0.181	**0.012**	0.215	0.517	0.306	0.444	0.107
β_2	0.888	0.412	0.659	0.972	0.984	0.772	0.274	0.439	0.964	0.518	0.512	0.953	0.144	0.064	0.739	0.352	0.671	0.891	0.537
**TREATMENT:VISIT**																			
δ	0.368	0.086	0.102	0.404	0.730	0.964	0.353	0.244	0.338	0.477	0.202	0.689	0.928	0.745	0.828	0.926	0.426	0.866	0.158
θ	0.568	0.287	0.899	0.176	0.498	0.812	0.475	0.226	0.526	0.607	0.647	0.680	0.755	0.854	0.443	0.792	0.630	0.798	0.176
α	0.536	0.093	0.581	0.320	0.133	0.276	0.060	0.262	0.636	0.776	0.512	0.470	0.362	0.281	0.312	0.501	**0.013**	0.839	0.333
β_1	0.827	0.395	0.950	0.228	0.786	0.517	0.980	0.648	0.480	0.112	0.362	0.422	0.726	0.111	0.216	0.504	0.060	0.057	0.805
β_2	0.135	**0.031**	0.334	0.060	0.617	0.789	0.211	0.279	0.315	0.305	0.644	0.473	0.697	0.652	0.823	0.215	0.742	0.451	0.674
**RESPONSE:TREATMENT:VISIT**																			
δ	0.978	0.981	0.575	0.460	0.974	0.213	0.748	0.595	0.564	0.709	0.725	0.737	0.713	0.191	0.865	0.532	0.687	0.299	0.261
θ	0.563	0.132	0.882	0.704	0.275	0.531	0.667	0.966	0.943	0.062	0.393	0.706	0.764	0.557	0.318	0.758	0.525	0.619	0.736
α	0.340	0.978	0.792	0.727	0.861	0.673	0.627	0.325	0.481	0.127	0.540	0.369	0.568	0.947	0.598	0.799	0.449	0.986	0.781
β_1	0.309	0.129	0.191	0.549	0.958	0.120	0.656	0.685	0.654	0.092	0.962	0.540	0.965	0.513	0.333	0.462	0.532	0.289	0.780
β_2	0.961	0.290	0.248	0.510	0.409	0.258	0.830	0.737	0.641	0.220	0.667	0.287	0.229	0.323	0.427	0.180	0.531	0.508	0.635

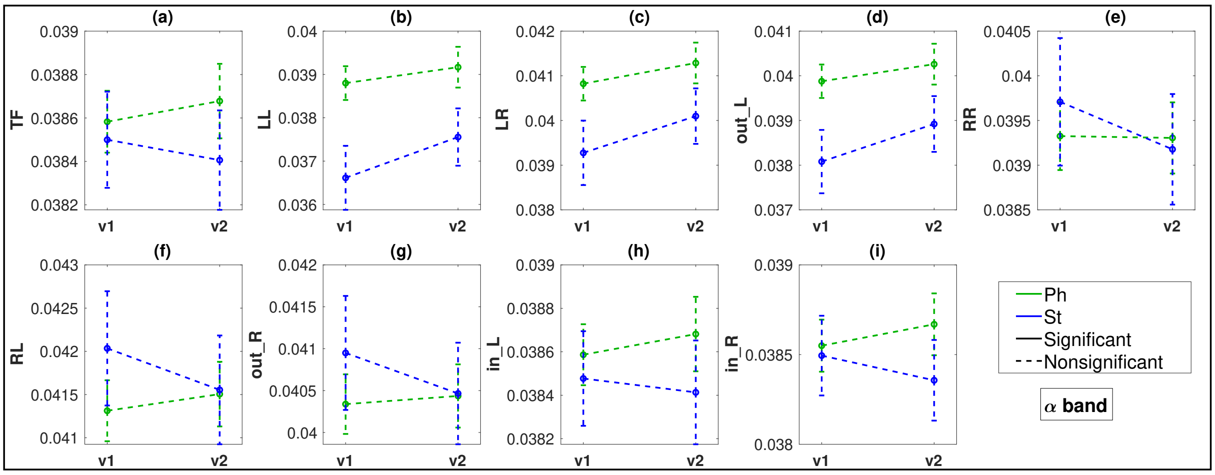

Figure 3: α band: FLOW Metric comparisons between visits 1 (v1) and 2 (v2) for the pharmacological (Ph) and neurostimulation (St) groups. Metrics with $p < 0.05$ plotted in bold lines and $p \geq 0.05$ are in dotted lines.

Table 4: **t-test, FDR-adjusted t-test, Signrank** p-values for the comparison of **FLOW metrics** in **visit 1** vs **visit 2** across different EEG bands for pharmacological and neurostimulation separately. Significant p values less than 0.05 are in bold font.

	**TF**	**LL**	**LR**	**out_L**	**RR**	**RL**	**out_R**	**in_L**	**in_R**
					**t-test**				
				**Pharmacological: visit 1 vs visit 2**					
δ	0.809	0.931	0.610	0.747	0.918	0.444	0.712	0.732	0.912
θ	**0.020**	0.277	**0.036**	0.091	0.500	0.085	0.196	**0.021**	**0.021**
α	0.478	0.448	0.314	0.411	0.960	0.600	0.796	0.474	0.386
β_1	0.744	0.446	0.504	0.510	0.101	0.081	0.082	0.598	0.967
β_2	**<0.001**	**0.043**	**0.016**	**0.024**	**0.010**	**0.049**	**0.021**	**<0.001**	**<0.001**
				**Neurostimulation: visit 1 vs visit 2**					
δ	**0.004**	**0.001**	**0.002**	**<0.001**	0.231	0.551	0.453	**0.005**	**0.007**
θ	0.748	0.073	0.056	0.053	0.457	0.512	0.516	0.815	0.920
α	0.536	0.128	0.118	0.129	0.370	0.390	0.394	0.691	0.394
β_1	0.766	0.508	0.376	0.384	0.241	0.425	0.317	0.700	0.994
β_2	**0.040**	0.106	0.205	0.127	0.277	0.287	0.291	0.100	0.056
				**FDR-adjusted t-test**					
				**Pharmacological: visit 1 vs visit 2**					
δ	0.931	0.931	0.931	0.931	0.931	0.931	0.931	0.931	0.931
θ	0.064	0.312	0.082	0.136	0.500	0.136	0.251	0.064	0.064
α	0.718	0.718	0.718	0.718	0.960	0.772	0.895	0.718	0.718
β_1	0.837	0.765	0.765	0.765	0.302	0.302	0.302	0.769	0.967
β_2	**<0.001**	**0.049**	**0.029**	**0.031**	**0.023**	**0.0497**	**0.031**	**<0.001**	**<0.001**
				**Neurostimulation: visit 1 vs visit 2**					
δ	**0.009**	**0.002**	**0.006**	**0.002**	0.297	0.551	0.510	**0.010**	**0.010**
θ	0.916	0.218	0.218	0.218	0.773	0.773	0.773	0.916	0.920
α	0.603	0.386	0.386	0.386	0.507	0.507	0.507	0.691	0.507
β_1	0.862	0.761	0.761	0.761	0.761	0.761	0.761	0.862	0.994
β_2	0.229	0.229	0.291	0.229	0.291	0.291	0.291	0.229	0.229
				**FDR-adjusted Permutation test:**					
				**Pharmacological: visit 1 vs visit 2**					
δ	0.929	0.929	0.929	0.929	0.929	0.929	0.929	0.929	0.929
θ	0.068	0.313	0.068	0.134	0.492	0.134	0.254	0.068	0.068
α	0.726	0.726	0.726	0.726	0.959	0.778	0.900	0.726	0.726
β_1	0.842	0.767	0.767	0.767	0.306	0.306	0.306	0.780	0.969
β_2	**<0.001**	**0.049**	**0.031**	**0.032**	**0.023**	**0.049**	**0.032**	**0.001**	**<0.001**
				**Neurostimulation: visit 1 vs visit 2**					
δ	**0.009**	**0.004**	**0.006**	**0.004**	0.292	0.558	0.508	**0.010**	**0.010**
θ	0.918	0.226	0.226	0.226	0.772	0.772	0.772	0.918	0.923
α	0.597	0.383	0.383	0.383	0.507	0.507	0.507	0.697	0.507
β_1	0.859	0.770	0.766	0.766	0.766	0.766	0.766	0.859	0.994
β_2	0.225	0.225	0.291	0.225	0.291	0.291	0.291	0.225	0.225

Table 5: **t-test, FDR-adjusted t-test, Signrank** p-values for the comparison of **INFLOW per channel** in **visit 1** vs **visit 2** across different EEG bands for pharmacological and neurostimulation separately. Significant p values less than 0.05 are in bold font.

	Fp1	F3	C3	P3	O1	F7	T3	T5	Fp2	F4	C4	P4	O2	F8	T4	T6	Fz	Cz	Pz
**t-test**																			
**Pharmacological: visit 1 vs visit 2**																			
δ	0.563	0.505	0.697	0.513	0.936	0.317	0.678	0.775	0.316	0.787	0.644	0.927	0.963	0.843	0.320	0.682	0.664	0.729	0.998
θ	0.067	**0.047**	**0.028**	**0.015**	**0.020**	**0.033**	0.061	**0.006**	0.052	**0.014**	**0.033**	**0.050**	**0.018**	**0.031**	**0.037**	**0.014**	**0.048**	**0.013**	**0.015**
α	0.479	0.419	0.642	0.411	0.425	0.602	0.516	0.993	0.289	0.324	0.585	0.411	0.457	0.310	0.538	0.899	0.763	0.827	0.957
β_1	0.516	0.865	0.599	0.264	0.970	1.000	0.774	0.454	0.927	0.265	0.466	0.602	0.771	0.141	0.759	0.428	0.533	0.545	0.550
β_2	0.200	**<0.001**	**0.009**	**<0.001**	**<0.001**	**0.041**	0.375	**0.005**	**<0.001**	**0.002**	**<0.001**	**<0.001**	**<0.001**	**0.005**	0.494	**<0.001**	**<0.001**	**<0.001**	**<0.001**
**Neurostimulation: visit 1 vs visit 2**																			
δ	**0.010**	**0.013**	**0.040**	**0.017**	0.100	0.114	**0.025**	**0.002**	0.121	**0.023**	0.104	**0.009**	**0.010**	0.124	0.054	**0.002**	**0.016**	**0.002**	**0.005**
θ	0.980	0.322	0.677	0.844	0.717	0.838	0.192	0.971	0.569	0.606	0.903	0.323	0.469	0.981	0.596	0.870	0.121	0.543	0.387
α	0.196	0.861	0.745	0.735	0.797	**0.048**	0.694	0.473	0.303	0.709	0.582	0.277	0.946	0.271	0.670	0.188	0.749	0.437	0.209
β_1	0.359	0.716	0.071	0.844	0.718	0.185	0.564	0.856	0.985	0.324	0.516	0.713	0.581	0.704	0.870	0.755	0.945	0.194	0.565
β_2	0.687	0.709	0.827	0.050	**0.008**	0.646	0.369	0.210	0.161	0.206	0.114	0.915	**0.048**	0.217	0.200	0.729	**0.012**	0.082	0.177
**FDR-adjusted t-test**																			
**Pharmacological: visit 1 vs visit 2**																			
δ	0.998	0.998	0.998	0.998	0.998	0.998	0.998	0.998	0.998	0.998	0.998	0.998	0.998	0.998	0.998	0.998	0.998	0.998	0.998
θ	0.067	0.058	0.052	**0.048**	**0.048**	0.052	0.065	**0.048**	0.058	**0.048**	0.052	0.058	**0.048**	0.052	0.054	**0.048**	0.058	**0.048**	**0.048**
α	0.871	0.871	0.871	0.871	0.871	0.871	0.871	0.993	0.871	0.871	0.871	0.871	0.871	0.871	0.871	0.993	0.967	0.982	0.993
β_1	0.953	1.000	0.953	0.953	1.000	1.000	0.980	0.953	1.000	0.953	0.953	0.953	0.980	0.953	0.980	0.953	0.953	0.953	0.953
β_2	0.224	**0.001**	**0.012**	**<0.001**	**<0.001**	**0.049**	0.396	**0.007**	**<0.001**	**0.002**	**0.001**	**<0.001**	**<0.001**	**0.007**	0.494	**<0.001**	**<0.001**	**0.002**	**0.001**
**Neurostimulation: visit 1 vs visit 2**																			
δ	**0.028**	**0.032**	0.058	**0.032**	0.124	0.124	**0.040**	**0.013**	0.124	**0.040**	0.124	**0.028**	**0.028**	0.124	0.073	**0.013**	**0.032**	**0.013**	**0.023**
θ	0.981	0.981	0.981	0.981	0.981	0.981	0.981	0.981	0.981	0.981	0.981	0.981	0.981	0.981	0.981	0.981	0.981	0.981	0.981
α	0.823	0.909	0.890	0.890	0.890	0.823	0.890	0.890	0.823	0.890	0.890	0.823	0.946	0.823	0.890	0.823	0.890	0.890	0.823
β_1	0.973	0.973	0.973	0.973	0.973	0.973	0.973	0.973	0.985	0.973	0.973	0.973	0.973	0.973	0.973	0.973	0.985	0.973	0.973
β_2	0.815	0.815	0.873	0.240	0.114	0.815	0.539	0.344	0.344	0.344	0.344	0.915	0.240	0.344	0.344	0.815	0.114	0.312	0.344
**FDR-adjusted Permutation test**																			
**Pharmacological: visit 1 vs visit 2**																			
δ	0.997	0.997	0.997	0.997	0.997	0.997	0.997	0.997	0.997	0.997	0.997	0.997	0.997	0.997	0.997	0.997	0.997	0.997	0.997
θ	0.065	0.056	**0.048**	**0.043**	**0.043**	**0.048**	0.063	**0.043**	0.056	**0.043**	**0.048**	0.056	**0.043**	**0.048**	**0.048**	**0.043**	0.056	**0.043**	**0.043**
α	0.870	0.870	0.880	0.870	0.870	0.870	0.870	0.992	0.870	0.870	0.870	0.870	0.870	0.870	0.870	0.992	0.959	0.988	0.992
β_1	0.951	1.000	0.951	0.951	1.000	1.000	0.979	0.951	1.000	0.951	0.951	0.951	0.979	0.951	0.979	0.951	0.951	0.951	0.951
β_2	0.227	**0.001**	**0.014**	**<0.001**	**<0.001**	**0.049**	0.396	**0.007**	**<0.001**	**0.003**	**0.001**	**0.001**	**<0.001**	**0.008**	0.504	**<0.001**	**<0.001**	**0.001**	**0.001**
**Neurostimulation: visit 1 vs visit 2**																			
δ	**0.031**	**0.031**	0.062	**0.035**	0.123	0.123	**0.039**	**0.014**	0.123	**0.039**	0.123	**0.031**	**0.031**	0.123	0.074	**0.014**	**0.031**	**0.014**	**0.026**
θ	0.982	0.982	0.982	0.982	0.982	0.982	0.982	0.982	0.982	0.982	0.982	0.982	0.982	0.982	0.982	0.982	0.982	0.982	0.982
α	0.843	0.911	0.887	0.887	0.887	0.843	0.887	0.887	0.843	0.887	0.887	0.843	0.949	0.843	0.887	0.843	0.887	0.887	0.843
β_1	0.974	0.974	0.974	0.974	0.974	0.974	0.974	0.974	0.985	0.974	0.974	0.974	0.974	0.974	0.974	0.974	0.985	0.974	0.974
β_2	0.821	0.821	0.873	0.254	0.111	0.821	0.545	0.340	0.340	0.340	0.340	0.915	0.254	0.340	0.340	0.821	0.111	0.301	0.340

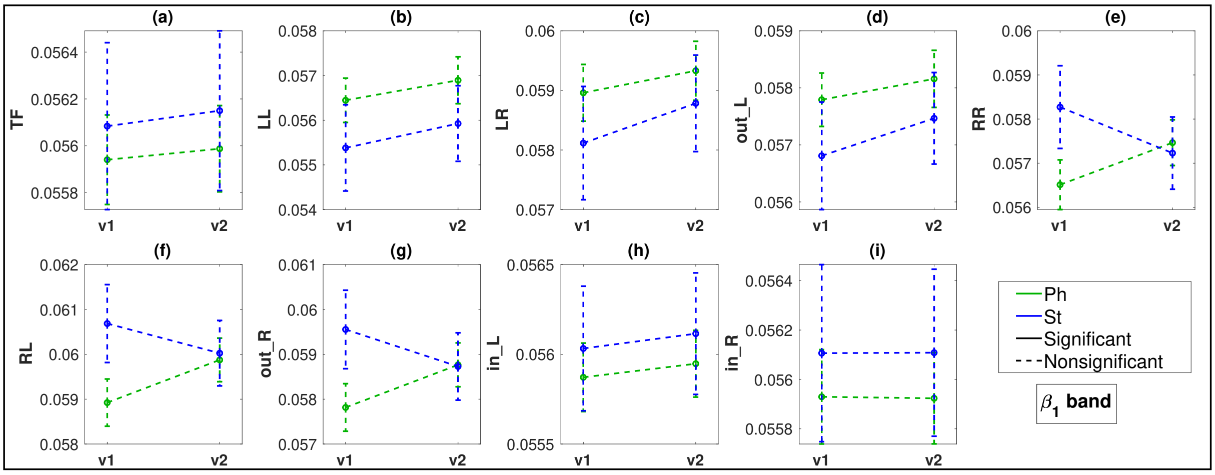

Figure 4: β_1 band: FLOW Metric comparisons between visits 1 (v1) and 2 (v2) for the pharmacological (Ph) and neurostimulation (St) groups. Metrics with $p < 0.05$ plotted in bold lines and $p \geq 0.05$ are in dotted lines.

Table 6: **t-test, FDR-adjusted t-test, ranksum** p-values for the comparison of the **FLOW metrics** at **visit 1** between **respondents (R)** and **nonrespondents (NR)** for the two treatment groups. Significant p-values less than 0.05 are in bold font.

	**TF**	**LL**	**LR**	**out_L**	**RR**	**RL**	**out_R**	**in_L**	**in_R**
					**t-test**				
					**Pharmacological: R visit 1 vs NR visit 1**				
$\boldsymbol{\delta}$	0.642	0.703	0.353	0.637	0.478	0.346	0.953	0.580	0.697
$\boldsymbol{\theta}$	0.288	**0.026**	**0.044**	**0.038**	0.876	0.799	0.767	0.344	0.279
$\boldsymbol{\alpha}$	**0.020**	0.818	0.535	0.675	**0.022**	**0.026**	**0.021**	**0.027**	**0.018**
$\boldsymbol{\beta_1}$	0.119	0.267	0.292	0.271	0.214	0.184	0.169	0.107	0.127
$\boldsymbol{\beta_2}$	0.387	0.974	0.749	0.860	0.609	0.542	0.591	0.291	0.472
					**Neurostimulation: R visit 1 vs NR visit 1**				
$\boldsymbol{\delta}$	**0.037**	0.531	0.343	0.465	0.364	0.288	0.197	**0.026**	**0.041**
$\boldsymbol{\theta}$	0.236	0.256	0.200	0.205	0.944	0.911	0.910	0.261	0.212
$\boldsymbol{\alpha}$	0.680	0.368	0.317	0.362	0.568	0.640	0.652	0.777	0.574
$\boldsymbol{\beta_1}$	0.749	0.071	**0.041**	0.053	0.140	0.265	0.204	0.883	0.756
$\boldsymbol{\beta_2}$	0.130	0.120	0.052	0.095	**0.032**	0.066	0.052	0.062	0.244
					**FDR-adjusted t-test**				
					**Pharmacological: R visit 1 vs NR visit 1**				
$\boldsymbol{\delta}$	0.790	0.790	0.790	0.790	0.790	0.790	0.953	0.790	0.790
$\boldsymbol{\theta}$	0.516	0.131	0.131	0.131	0.876	0.876	0.876	0.516	0.516
$\boldsymbol{\alpha}$	**0.040**	0.818	0.688	0.760	**0.040**	**0.040**	**0.040**	**0.040**	**0.040**
$\boldsymbol{\beta_1}$	0.292	0.292	0.292	0.292	0.292	0.292	0.292	0.292	0.292
$\boldsymbol{\beta_2}$	0.914	0.974	0.963	0.968	0.914	0.914	0.914	0.914	0.914
					**Neurostimulation: R visit 1 vs NR visit 1**				
$\boldsymbol{\delta}$	0.123	0.531	0.468	0.523	0.468	0.468	0.443	0.123	0.123
$\boldsymbol{\theta}$	0.391	0.391	0.391	0.391	0.944	0.944	0.944	0.391	0.391
$\boldsymbol{\alpha}$	0.765	0.765	0.765	0.765	0.765	0.765	0.765	0.777	0.765
$\boldsymbol{\beta_1}$	0.851	0.212	0.212	0.212	0.316	0.398	0.367	0.883	0.851
$\boldsymbol{\beta_2}$	0.146	0.146	0.119	0.142	0.119	0.119	0.119	0.119	0.244
					**FDR-adjusted Permutation test**				
					**Pharmacological: R visit 1 vs NR visit 1**				
$\boldsymbol{\delta}$	0.790	0.790	0.790	0.790	0.790	0.790	0.952	0.790	0.790
$\boldsymbol{\theta}$	0.513	0.135	0.135	0.135	0.876	0.876	0.876	0.513	0.513
$\boldsymbol{\alpha}$	**0.042**	0.820	0.694	0.762	**0.042**	**0.042**	**0.042**	**0.042**	**0.042**
$\boldsymbol{\beta_1}$	0.293	0.293	0.293	0.293	0.293	0.293	0.293	0.293	0.293
$\boldsymbol{\beta_2}$	0.913	0.970	0.962	0.969	0.913	0.913	0.913	0.913	0.913
					**Neurostimulation: R visit 1 vs NR visit 1**				
$\boldsymbol{\delta}$	0.131	0.538	0.471	0.528	0.471	0.471	0.444	0.131	0.131
$\boldsymbol{\theta}$	0.395	0.395	0.395	0.395	0.948	0.948	0.948	0.395	0.395
$\boldsymbol{\alpha}$	0.760	0.760	0.760	0.760	0.760	0.760	0.760	0.773	0.760
$\boldsymbol{\beta_1}$	0.849	0.207	0.207	0.207	0.315	0.399	0.366	0.885	0.849
$\boldsymbol{\beta_2}$	0.144	0.144	0.128	0.144	0.128	0.128	0.128	0.128	0.246

Table 7: **t-test, FDR-adjusted t-test, ranksum** p-values for the comparison of the **INFLOW per channel metrics** at **visit 1** between **respondents (R)** and **nonrespondents (NR)** for the two treatment groups. Significant p-values less than 0.05 are in bold font.

	Fp1	F3	C3	P3	O1	F7	T3	T5	Fp2	F4	C4	P4	O2	F8	T4	T6	Fz	Cz	Pz
**t-test**																			
**Pharmacological: R visit 1 vs NR visit 1**																			
δ	0.560	0.683	0.298	0.408	0.604	0.621	0.648	0.614	0.574	0.687	0.386	0.542	0.663	0.630	0.988	0.276	0.932	0.685	0.456
θ	0.423	0.336	0.287	0.452	0.252	0.479	0.381	0.275	0.314	0.304	0.219	0.315	0.326	0.353	0.279	0.228	0.244	0.251	0.130
α	**0.015**	0.062	**0.033**	**0.035**	0.113	**0.003**	0.311	0.061	**0.028**	**0.013**	**0.034**	**0.007**	**0.008**	**0.030**	0.325	0.083	**0.034**	0.068	**0.010**
β_1	**0.035**	0.223	0.366	0.080	0.312	0.232	0.064	**0.034**	0.071	0.431	0.142	0.179	0.172	0.220	0.152	0.051	0.324	0.215	0.074
β_2	0.199	0.169	0.351	0.930	0.650	0.176	0.479	0.695	0.160	0.351	0.931	0.590	0.990	0.261	0.809	0.810	0.410	0.790	0.500
**Neurostimulation: R visit 1 vs NR visit 1**																			
δ	**0.012**	0.099	0.081	0.121	0.122	0.061	**0.022**	**0.019**	0.068	0.371	**0.004**	0.059	**0.039**	0.113	0.095	0.100	0.134	0.118	0.133
θ	0.258	0.164	0.223	0.400	0.224	0.304	0.220	0.412	0.182	0.180	0.177	0.172	0.281	0.253	0.273	0.254	0.410	0.305	0.114
α	0.808	0.645	0.925	0.362	0.770	0.794	0.445	0.905	0.434	0.564	0.896	0.620	0.583	0.234	0.820	0.939	0.501	0.665	0.526
β_1	0.893	0.854	0.699	0.967	0.835	0.906	0.625	0.863	0.807	0.961	0.915	0.782	0.515	0.461	0.944	0.610	0.618	0.412	0.404
β_2	0.363	0.134	0.405	0.183	0.338	**0.041**	**0.018**	0.447	0.289	**0.020**	0.098	0.546	0.291	0.452	0.741	0.296	0.297	0.249	0.254
**FDR-adjusted t-test**																			
**Pharmacological: R visit 1 vs NR visit 1**																			
δ	0.767	0.767	0.767	0.767	0.767	0.767	0.767	0.767	0.767	0.767	0.767	0.767	0.767	0.767	0.988	0.767	0.984	0.767	0.767
θ	0.473	0.447	0.447	0.478	0.447	0.479	0.453	0.447	0.447	0.447	0.447	0.447	0.447	0.447	0.447	0.447	0.447	0.447	0.447
α	**0.048**	0.084	0.055	0.055	0.126	**0.048**	0.325	0.084	0.055	**0.048**	0.055	**0.048**	**0.048**	0.055	0.325	0.099	0.055	0.086	**0.048**
β_1	0.218	0.294	0.387	0.218	0.362	0.294	0.218	0.218	0.218	0.431	0.294	0.294	0.294	0.294	0.294	0.218	0.362	0.294	0.218
β_2	0.943	0.943	0.943	0.983	0.961	0.943	0.943	0.961	0.943	0.943	0.983	0.961	0.994	0.943	0.961	0.961	0.943	0.961	0.943
**Neurostimulation: R visit 1 vs NR visit 1**																			
δ	0.106	0.142	0.142	0.142	0.142	0.142	0.106	0.106	0.142	0.371	0.082	0.142	0.142	0.142	0.142	0.142	0.142	0.142	0.142
θ	0.363	0.363	0.363	0.412	0.363	0.363	0.363	0.412	0.363	0.363	0.363	0.363	0.363	0.363	0.363	0.363	0.412	0.363	0.363
α	0.939	0.939	0.939	0.939	0.939	0.939	0.939	0.939	0.939	0.939	0.939	0.939	0.939	0.939	0.939	0.939	0.939	0.939	0.939
β_1	0.967	0.967	0.967	0.967	0.967	0.967	0.967	0.967	0.967	0.967	0.967	0.967	0.967	0.967	0.967	0.967	0.967	0.967	0.967
β_2	0.492	0.471	0.505	0.471	0.492	0.259	0.192	0.505	0.471	0.192	0.465	0.576	0.471	0.505	0.741	0.471	0.471	0.471	0.471
**FDR-adjusted Permutation test**																			
**Pharmacological: R visit 1 vs NR visit 1**																			
δ	0.770	0.770	0.770	0.770	0.770	0.770	0.770	0.770	0.770	0.770	0.770	0.770	0.770	0.770	0.990	0.770	0.986	0.770	0.770
θ	0.474	0.439	0.439	0.476	0.439	0.488	0.447	0.439	0.439	0.439	0.439	0.439	0.439	0.439	0.439	0.439	0.439	0.439	0.439
α	**0.041**	0.084	0.056	0.056	0.125	**0.041**	0.323	0.084	0.056	**0.041**	0.056	**0.041**	**0.041**	0.056	0.323	0.096	0.056	0.087	**0.041**
β_1	0.222	0.296	0.389	0.222	0.363	0.296	0.222	0.222	0.222	0.425	0.296	0.296	0.296	0.296	0.296	0.222	0.363	0.296	0.222
β_2	0.949	0.949	0.949	0.982	0.968	0.949	0.949	0.968	0.949	0.949	0.982	0.968	0.993	0.949	0.968	0.968	0.949	0.968	0.949
**Neurostimulation: R visit 1 vs NR visit 1**																			
δ	0.098	0.144	0.144	0.144	0.144	0.144	0.098	0.098	0.144	0.369	0.070	0.144	0.143	0.144	0.144	0.144	0.144	0.144	0.144
θ	0.365	0.365	0.365	0.421	0.365	0.365	0.365	0.421	0.365	0.365	0.365	0.365	0.365	0.365	0.365	0.365	0.421	0.365	0.365
α	0.937	0.937	0.937	0.937	0.937	0.937	0.937	0.937	0.937	0.937	0.937	0.937	0.937	0.937	0.937	0.937	0.937	0.937	0.937
β_1	0.965	0.965	0.965	0.965	0.965	0.965	0.965	0.965	0.965	0.965	0.965	0.965	0.965	0.965	0.965	0.965	0.965	0.965	0.965
β_2	0.489	0.477	0.498	0.477	0.489	0.248	0.173	0.498	0.477	0.173	0.477	0.573	0.477	0.498	0.744	0.477	0.477	0.477	0.477

Table 8: Pearson correlation analyses between baseline causality global FLOW metrics in the α band and proportional symptom improvement in the pharmacological treatment group. Bootstrap confidence intervals (CI) computed with 5000 resamples. P-values from two-sided permutation tests (5000 permutations); padj from FDR adjustment. Significant padj values less than 0.05 are in bold font.

Metric	Correlation (r)	CI Low	CI High	p-value	padj
**Pearson**					
**TF**	-0.239	-0.392	-0.076	0.006	**0.013**
**LL**	0.007	-0.168	0.185	0.935	0.993
**LR**	-0.015	-0.203	0.170	0.874	0.993
**out_L**	0.001	-0.178	0.187	0.993	0.993
**RR**	-0.229	-0.374	-0.077	0.007	**0.013**
**RL**	-0.222	-0.368	-0.071	0.013	**0.019**
**out_R**	-0.234	-0.376	-0.082	0.007	**0.013**
**in_L**	-0.232	-0.381	-0.066	0.006	**0.013**
**in_R**	-0.242	-0.387	-0.083	0.005	**0.013**

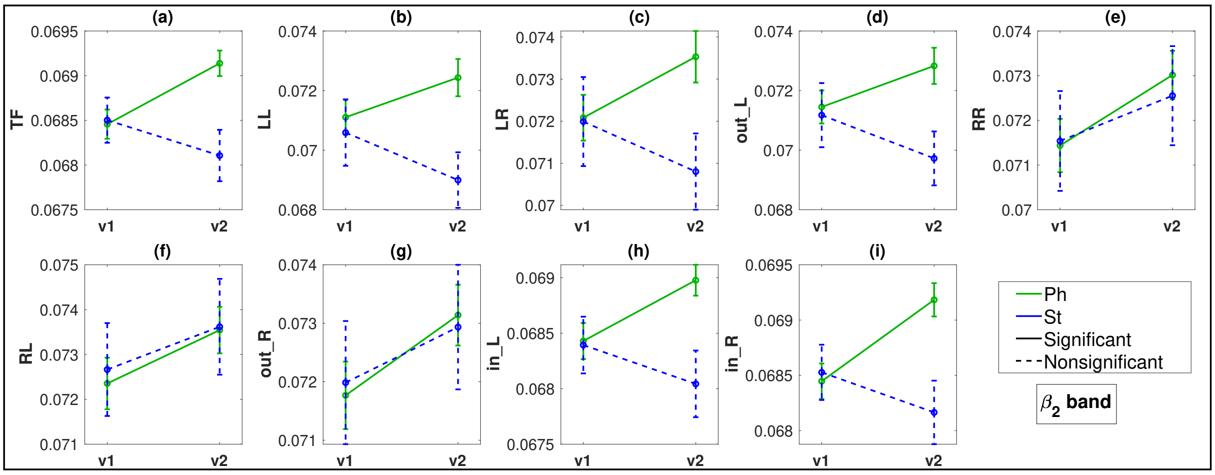

Figure 5: β_2 band: FLOW Metric comparisons between visits 1 (v1) and 2 (v2) for the pharmacological (Ph) and neurostimulation (St) groups. Metrics with $p < 0.05$ plotted in bold lines and $p \geq 0.05$ are in dotted lines.

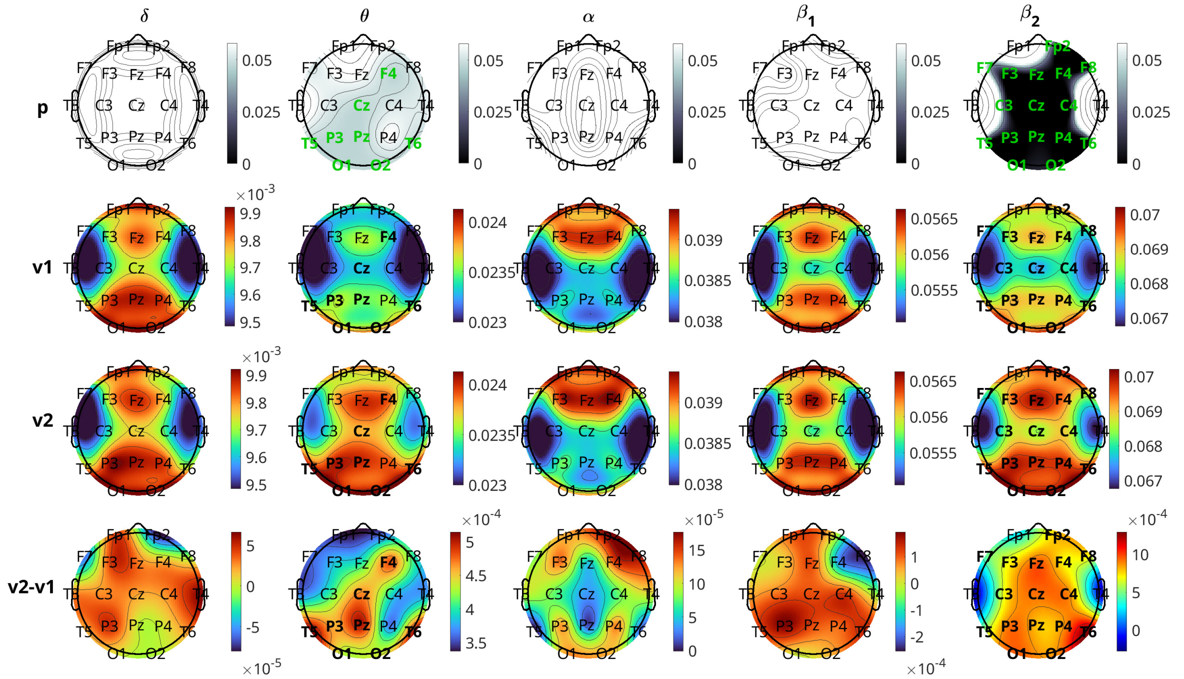

Figure 6: Brain topographical plots for the comparison of inflow per channel across the two visits 1 (v1) and 2 (v2), for the pharmacological group. The first row of panels is the p-values for the comparison. The 2nd and 3rd rows present the average values (across subjects) of the inflow for each visit, while the 4th row presents the difference. Columns correspond to frequency bands, as indicated by the text above the top panels. Channels with p-values < 0.05 are indicated in green-coloured text in the first row.

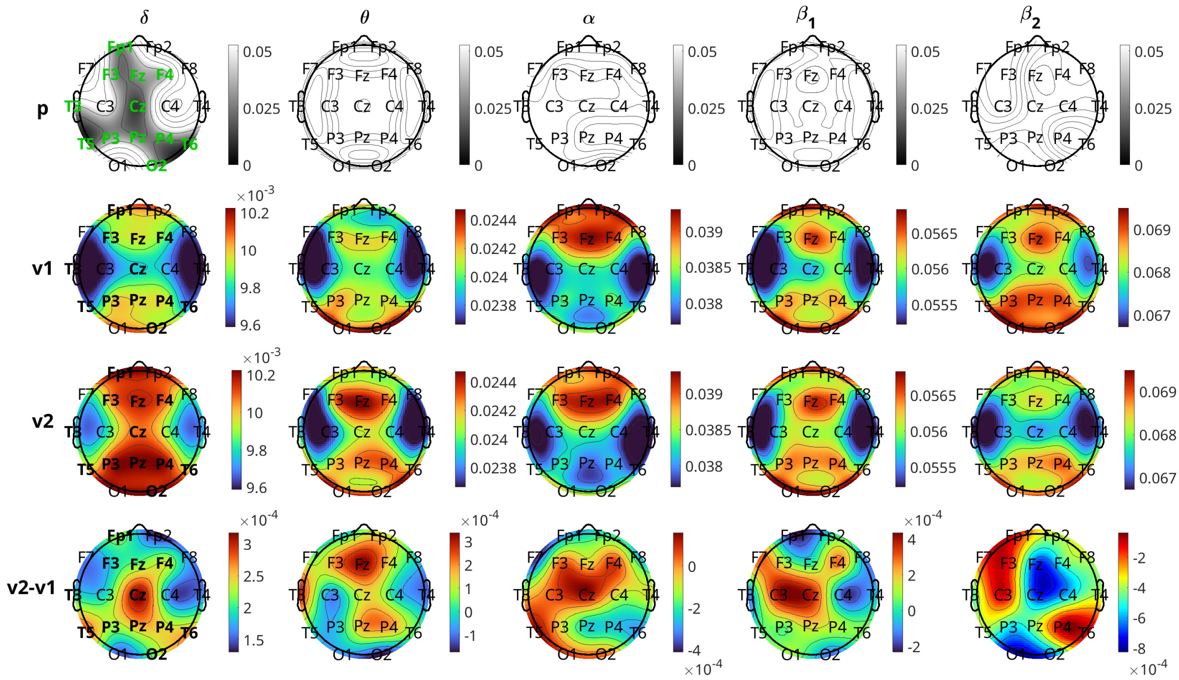

Figure 7: Brain topographical plots for the comparison of inflow per channel across the two visits 1 (v1) and 2 (v2), for the neurostimulation group. The first row of panels is the p-values for the comparison. The 2nd and 3rd rows present the average values (across subjects) of the inflow for each visit, while the 4th row presents the difference. Columns correspond to frequency bands, as indicated by the text above the top panels. Channels with p-values < 0.05 are indicated in green-coloured text in the first row.

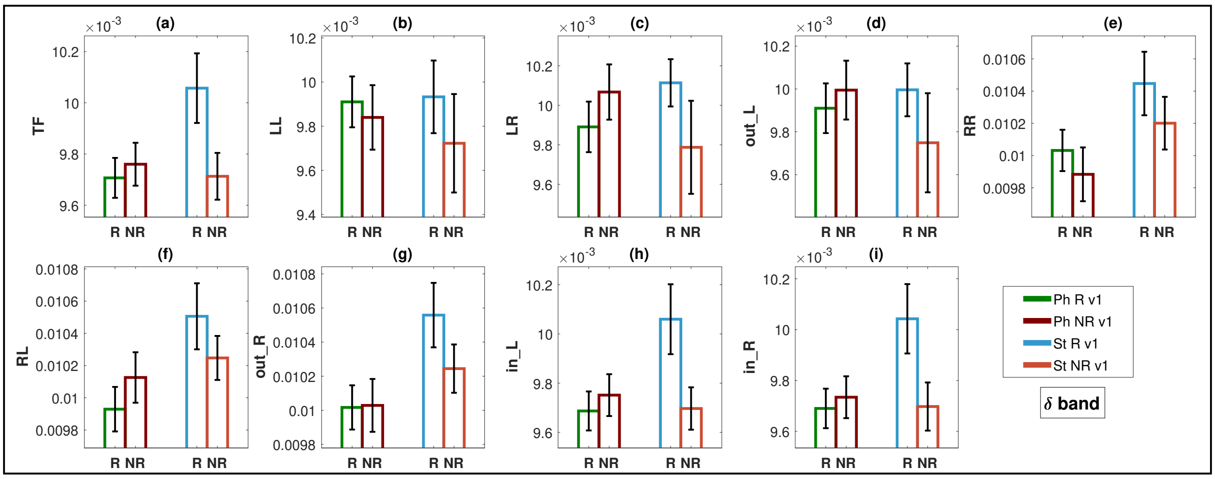

Figure 8: δ band: Bar plots representing the FLOW metrics at the time of visit 1 for respondents (R) and nonrespondents (NR) to pharmacological (Ph) and neurostimulation (St) treatment. Metrics with $p < 0.05$ have face colours and for $p \geq 0.05$ have no face colours in the bar plots. The first two bar plots in each panel are for Ph and the next two are for St.

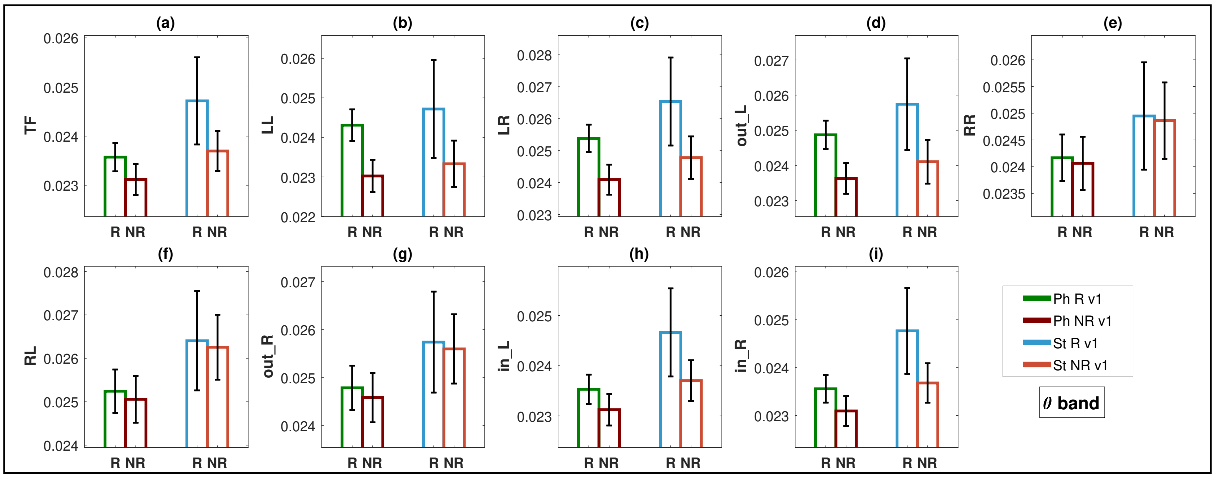

Figure 9: θ band: Bar plots representing the FLOW metrics at the time of visit 1 for respondents (R) and nonrespondents (NR) to pharmacological (Ph) and neurostimulation (St) treatment. Metrics with $p < 0.05$ have face colours and for $p \geq 0.05$ have no face colours in the bar plots. The first two bar plots in each panel are for Ph and the next two are for St.

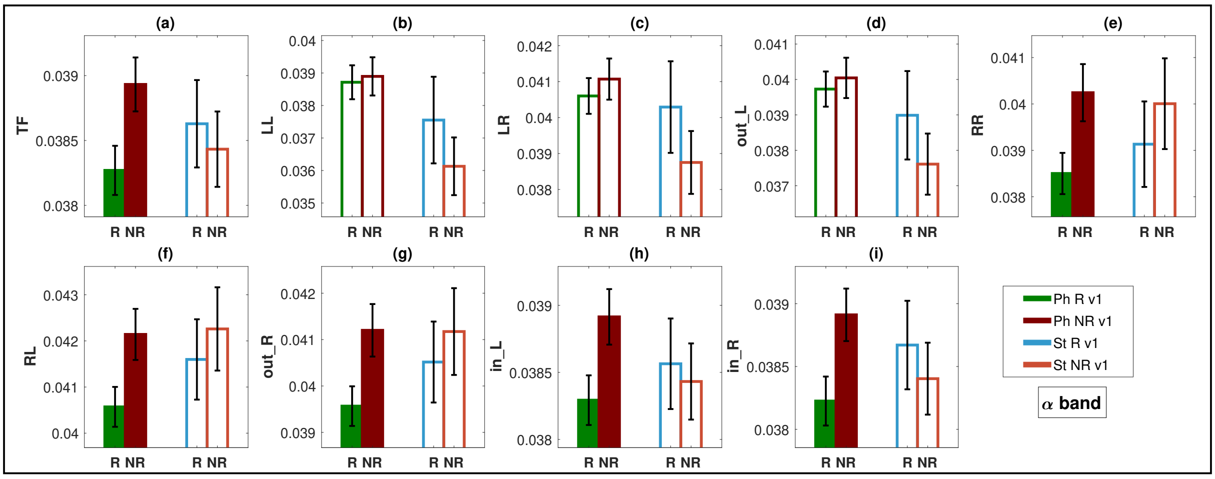

Figure 10: α band: Bar plots representing the FLOW metrics at the time of visit 1 for respondents (R) and nonrespondents (NR) to pharmacological (Ph) and neurostimulation (St) treatment. Metrics with $p < 0.05$ have face colours and for $p \geq 0.05$ have no face colours in the bar plots. The first two bar plots in each panel are for Ph and the next two are for St.

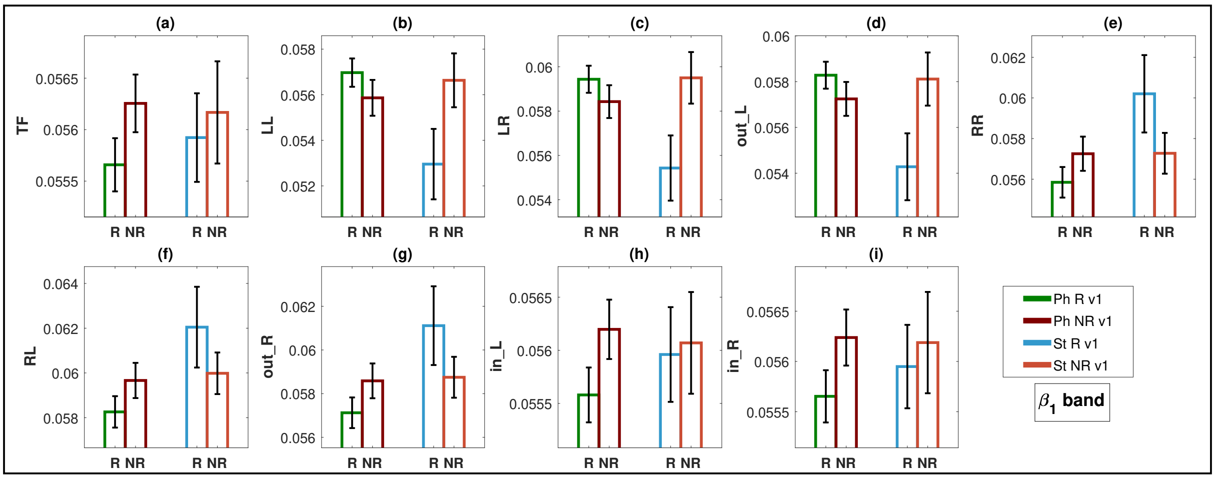

Figure 11: β_1 band: Bar plots representing the FLOW metrics at the time of visit 1 for respondents (R) and nonrespondents (NR) to pharmacological (Ph) and neurostimulation (St) treatment. Metrics with $p < 0.05$ have face colours and for $p \geq 0.05$ have no face colours in the bar plots. The first two bar plots in each panel are for Ph and the next two are for St.

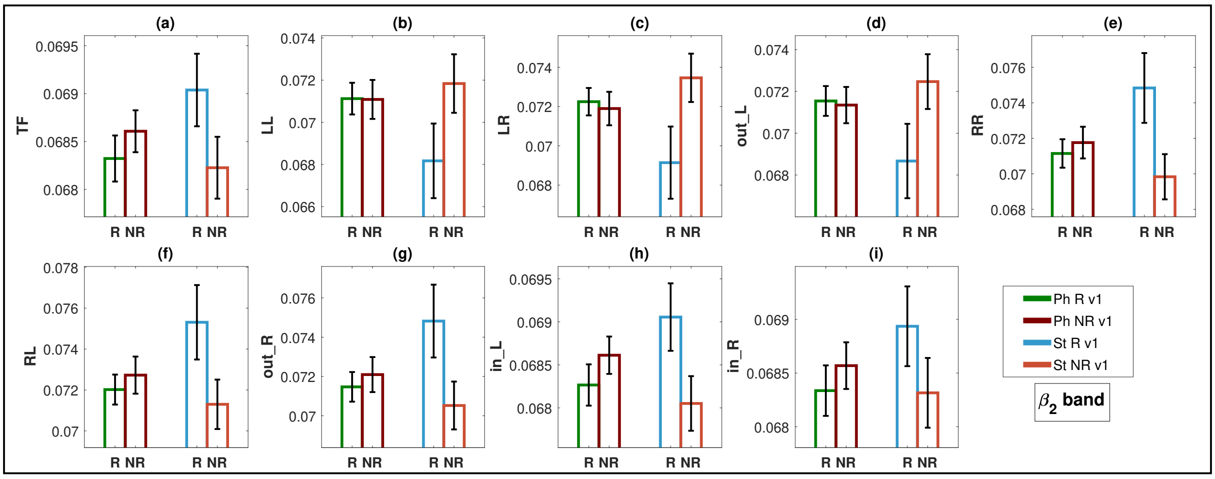

Figure 12: β_2 band: Bar plots representing the FLOW metrics at the time of visit 1 for respondents (R) and nonrespondents (NR) to pharmacological (Ph) and neurostimulation (St) treatment. Metrics with $p < 0.05$ have face colours and for $p \geq 0.05$ have no face colours in the bar plots. The first two bar plots in each panel are for Ph and the next two are for St.

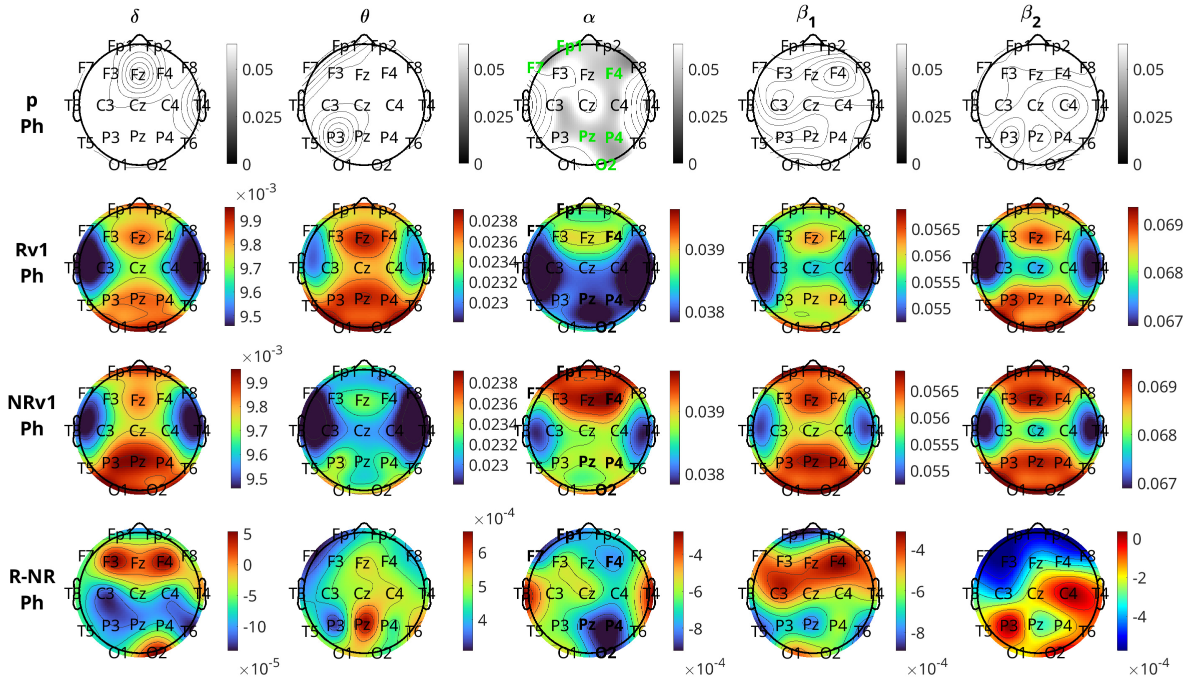

Figure 13: Brain topographical plots for the comparison of inflow per channel between respondents (R) and nonrespondents (NR) for the pharmacological (Ph) group at visit 1 (v1). The first row of panels is the p-values. The second and the third rows are the average inflow per channel (across all subjects) at visit 1 for respondents (R) and nonrespondents (NR). The fourth row is the difference in inflow between R and NR averaged over all subjects. Columns correspond to frequency bands, as indicated by the text above the top panels. Channels with p-values < 0.05 are indicated in green-coloured text in the 1st and 3rd rows.

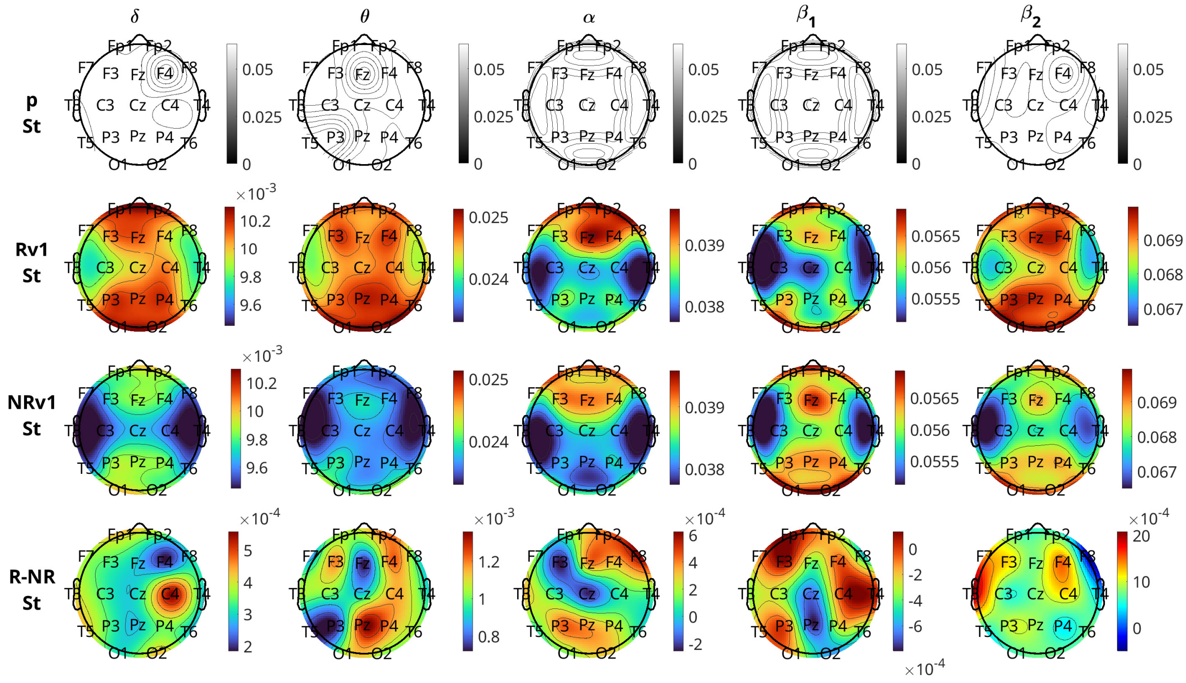

Figure 14: Brain topographical plots for the comparison of inflow per channel between respondents (R) and nonrespondents (NR) for the neurostimulation (St) group at visit 1 (v1). The first row of panels is the p-values. The second and the third rows are the average inflow per channel (across all subjects) at visit 1 for respondents (R) and nonrespondents (NR). The fourth row is the difference in inflow between R and NR averaged over all subjects. Columns correspond to frequency bands, as indicated by the text above the top panels. Channels with p-values < 0.05 are indicated in green-coloured text in the 1st and 3rd rows.
